# The roles of physician associates and advanced nurse practitioners in the National Health Service in the UK: a scoping review and narrative synthesis

**DOI:** 10.1186/s12960-022-00766-5

**Published:** 2022-09-15

**Authors:** Hanyu Wang, Mike English, Samprita Chakma, Mesulame Namedre, Elaine Hill, Shobhana Nagraj

**Affiliations:** 1 Nuffield Department of Medicine, Oxford Centre for Global Health Research, University of Oxford, Oxford, UK; 2 KEMRI-Wellcome Trust Research Programme, KEMRI-Wellcome Trust, Nairobi, Kenya; 3 Independent Researcher, Suva, Fiji; 4 Oxford University Hospitals NHS Foundation Trust, Oxford, UK

**Keywords:** Non-physician clinician, Physician associate, Advanced nurse practitioners, National Health Service, Task shifting

## Abstract

**Background:**

Mid-level practitioners (MLPs), including physician associates (PAs) and advanced nurse practitioners (ANPs), have emerged to address workforce shortages in the UK and perform specific roles in relation to population needs. This has resulted in new ways of working and changes to established professional hierarchies. We conducted a study to investigate the career development, competencies, effectiveness, perceptions, and regulation of PAs and ANPs, with the aim of understanding ways to effectively integrate MLPs into the NHS workforce.

**Methods:**

We conducted a systematic scoping review following PRISMA guidelines. Embase, Medline, the Cochrane database, Pubmed, and CINAHL databases were searched, using terms relating to PAs and ANPs in the UK. A total of 128 studies (60 on PAs and 68 on ANPs) were included in the final analysis. A narrative synthesis, guided by the pre-defined themes and emerging themes, was conducted to bring together the findings.

**Results:**

PAs are educated on a medical model with basic medical skills but lack formal professional regulation and do not have prescribing rights. ANPs are educated on a nurse model with enhanced skills that depend on roles within specific specialities, and their governance is mostly employer-led. PAs are primarily employed in secondary care. ANPs are employed widely in both primary and secondary care. No defined career progression exists for PAs. In contrast, becoming an ANP is a form of career progression within nursing. Both roles were regarded as cost-effective in comparison to doctors performing simple tasks. PAs were less understood compared to ANPs and received a mixed reception from colleagues, which sometimes undermined their professional identity, whereas ANPs were mostly welcomed by colleagues.

**Conclusions:**

Potential ways to better integrate PAs and ANPs into the NHS workforce include further initiatives by regulatory bodies and the NHS to create more awareness and clearer role definitions for MLPs, outline potential for career progression, offer transparency with regard to remuneration, and introduction of prescribing rights. Future research might include more cadres of MLPs and explore the international literature.

**Supplementary Information:**

The online version contains supplementary material available at 10.1186/s12960-022-00766-5.

## Introduction

Healthcare systems globally are experiencing a shortage of physicians. Although the largest deficits of healthcare workers are in low and middle-income countries, high-income countries, including the UK, are also experiencing severe workforce shortages [1, 2]. In 2019–2020, the National Health Service (NHS) spent approximately £56.1 billion (46.6% of its total budget) on workforce salaries [3]. The combination of medical workforce shortages and increasing financial costs has urged many countries to develop more cost-effective models of healthcare, for example, through shortening the duration of training of healthcare personnel with more generalist skills compared to specialist physicians [4]. In the UK, mid-level practitioners (MLPs), including physician associates (PAs) and advanced nurse practitioners (ANPs) have been deployed to task-share with physicians [5, 6].

ANPs were introduced within the NHS in the 1980s in response to the shortage of junior doctors [7, 8]. ANPs are registered nurses who work at an advanced level of practice. The ANP role is firmly rooted in the philosophy of nursing practice and extends to include clinical competencies traditionally expected of doctors [9]. The Royal College of Nursing (RCN) recommended that ANPs are educated at a Masters level with expert clinical knowledge and skills [10], but in practice some ANPs do not have an advanced degree [8, 11].

Similarly, in the early 2000s, a shortage of nurses and doctors in the UK prompted the Department of Health (DoH) to develop new roles through its Changing Workforce Programme [12]. PAs have a long history in the US, dating back to the 1960s [13]. The first group of PAs, trained in the US, were introduced to the UK in 2003 to address workforce shortages in underserved primary care practices in the West Midlands [13]. Unlike ANPs, PAs are dependent practitioners, trained in the medical model to work as part of a multidisciplinary team, as generalist healthcare professionals [6]. The number of PA educational programs have rapidly expanded over the past decade [13, 14], and in 2021, around 2500 qualified PAs were working in the UK, with a projected growth of 1000 per year [15, 16]. Thus, the profession will start to be a significant part of the future NHS workforce.

The addition of both PAs and ANPs within the NHS has resulted in task-sharing between medical and nursing professions, which can challenge established professional boundaries and hierarchies [17, 18]. As the number of PAs grows rapidly, it is important to understand the similarities and differences between this relatively new profession (PAs) and the comparatively well-established ANP role. This might help to incorporate and integrate PAs and, potentially, future new MLPs into the NHS workforce. With this long-term aim in mind, we undertook a systematic scoping review of the literature and narrative synthesis to explore the career development, competency, effectiveness, perceptions, and regulation of PAs and ANPs in the UK.

## Methods

### Search strategy and study selection

Our scoping review was based on the methodology outlined by Peters et al. [19]. We conducted a structured search in Embase (1974–Sep 2021), Medline (1974–Sep 2021), PubMed (1996–Sep 2021), CINAHL (1984–Sep 2021), and Cochrane Database (1993–Sep 2021) to identify the literature on PAs and ANPs in the UK. Briefly, the search strategy consists of two parts: (i) terms describing PAs or ANPs, AND (ii) terms describing the UK. The search strategy is detailed in Additional file 1: Appendix 1.

The review was conducted using PRISMA guidelines [20]. Studies were imported into *Endnote X9*. After duplicate studies were removed, we conducted two rounds of screening. In the first round, titles and abstracts were read and studies were excluded if their topics were not relevant. This was followed by a full-text screening. Studies were excluded in the second-round if: (1) their topics are not directly relevant to PAs or ANPs; (2) not relevant to the UK; (3) designs are not appropriate, e.g., letters, commentary, conference presentations. Included papers were journal articles, written in English, exploring any aspect of PAs or/and ANPs in the UK.

### Data extraction and synthesis

A narrative synthesis was conducted to bring together the findings from the selected literature [21]. The reviewing and the narrative synthesis processes were guided by the pre-defined research themes, including the career development, competency, effectiveness, perceptions, and regulation of PAs and ANPs. New themes that emerged and were deemed relevant to our aims were also included. NVivo 12 (*QSR International, Doncaster, Australia*) was used to curate the data for qualitative coding. Selected studies were first imported into NVivo and coded according to the themes by a single investigator. The investigator then extracted the statements from the literature into a data abstraction sheet for result summarisation.

## Results

### Literature selection results

**Fig. 1:**
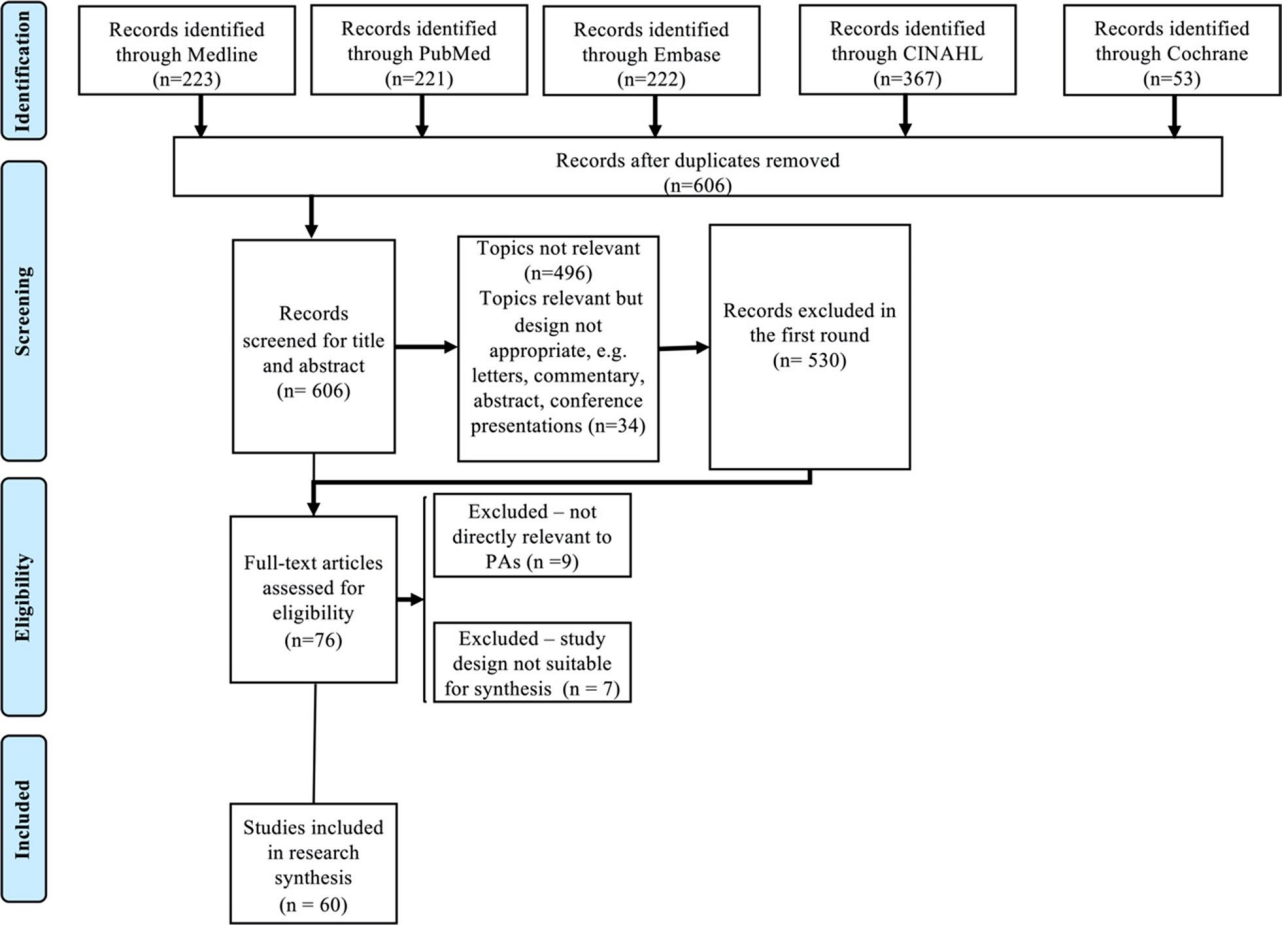
PRISMA [20] diagram for PAs

**Fig. 2:**
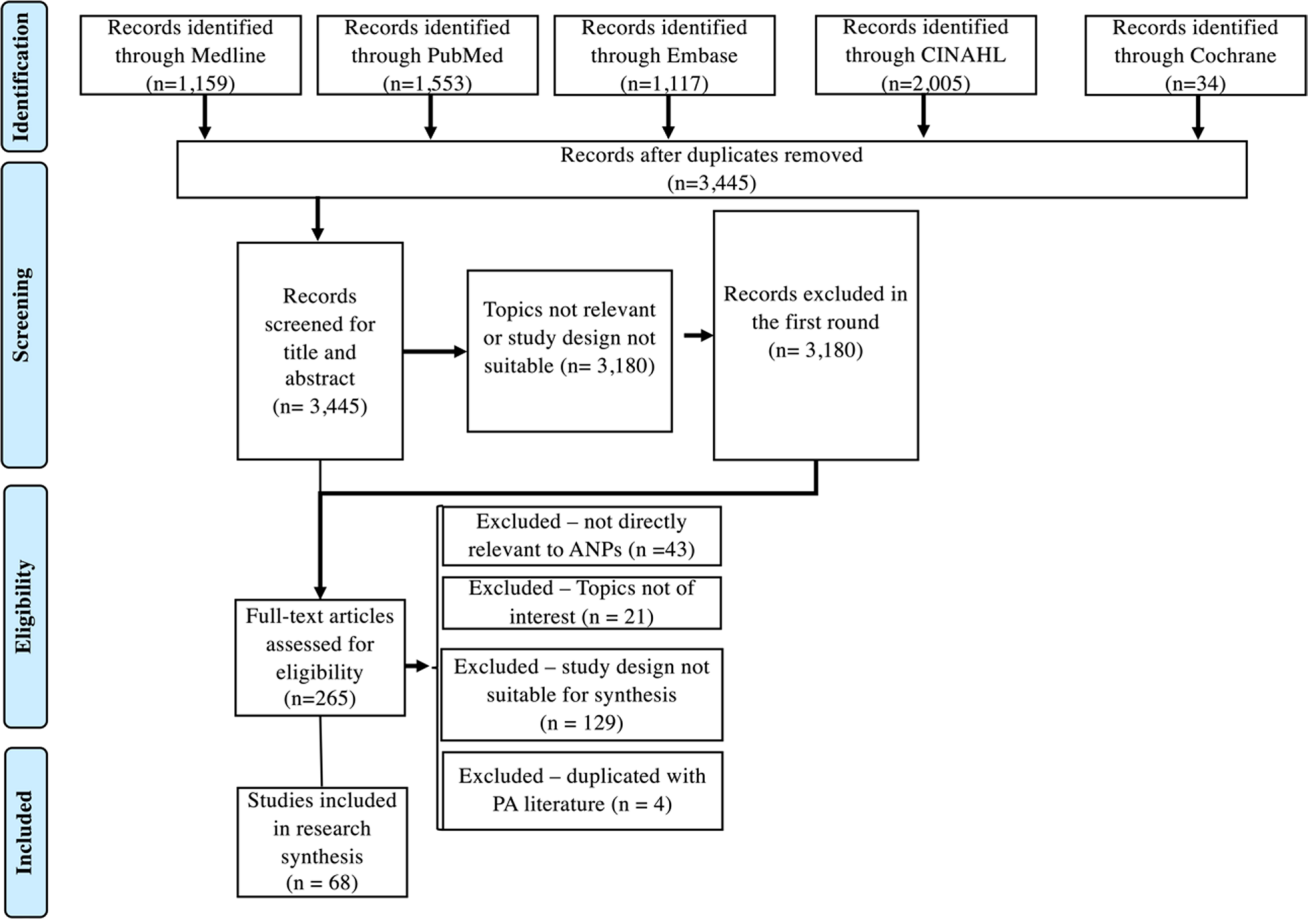
PRISMA [20] diagram for ANPs

The literature selection process is summarised in Figs. 1 and 2. For PAs, a total of 606 were identified from the electronic databases after removing duplicated articles. After screening for title and abstracts, 530 articles were excluded, and 76 studies were screened for full text. After excluding irrelevant articles or articles with unsuitable study designs for data synthesis, a total of 60 studies were included in the scoping review for PAs. For ANPs, the initial search identified a total of 3,445 articles after removing duplicated articles. After screening for title and abstracts, 265 articles were included for full-text screening. After excluding irrelevant articles, articles with unsuitable study designs for data synthesis, and duplicated literature with PAs, a total of 68 studies were included in the scoping review for ANPs.

### Overview of the selected studies

Overview of the included 60 studies for PAs and 68 studies for ANPs are presented in Additional file 2: Appendix 2 and Additional file 3: Appendix 3, respectively. The first included article on PAs in the UK was published in 1980 and the majority (45 out of 60 studies) were published after 2010. Of the 60 included PA articles, 17 studies were qualitative studies, 15 used quantitative data, 18 articles were reviews, 6 were mixed-methods studies, and 4 were editorials and a report of qualitative studies. For the 68 studies on ANPs, 11 studies were published before 2000 and more than half of the studies were published after 2010 (36 out of 68). Thirty of the 68 studies used qualitative methods, 19 studies were reviews, 11 studies were quantitative, 7 studies employed a mixed-methods design, and one was a randomised controlled trial. All included studies were peer-reviewed publications.

### Summary of main results

The narrative synthesis of the literature from the scoping review generated insights on the competencies, career development, effectiveness, perceptions, and regulation of PAs in comparison to ANPs. The major findings around those five themes and the similarities and differences between PAs and ANPs are summarised in Table 1.

**Table 1:** Similarities and differences on the major themes regarding PAs and ANPs

Sub-themes	Similarities	Differences
Career		
Employment	• Both see employment demand rising • Job content depends on specific posts and role definition is unclear	• PAs are primarily employed in secondary care, compared to the majority of ANPs in primary care • PAs have less variation in titles compared to ANPs
Career progression	• No clear career progression for both roles	• ANPs emerged from nurses so they are already at an advanced level in career development, whereas it is not the case for PAs
Job satisfaction	• Both are generally satisfied with their roles	• PAs encounter more issues with lack of recognition and lack of prescribing rights
Competency		
Education	• Both are recommended to be educated to a master’s level or equivalent	• PAs are educated on a medical model, whereas ANPs are on a nursing model •PAs generally have a master’s degree, whereas for ANPs master’s degree is recommended but not essential
Key skills	• Both have a mixture of nursing and medical skills	• ANPs’ skill sets are nursing-oriented, whereas PAs are medicine-oriented •Skills for ANPs depend more on the specific specialities, whereas PAs are educated to a similar level
Effectiveness		
Patient outcomes	• In simple tasks (minor illnesses), patient outcomes are comparable to GPs in primary care for both roles	• In secondary care, PAs have similar patient outcomes as FY2 doctors, whereas no evidence is identified for ANPs in secondary care
Cost-effectiveness	• In simple tasks, there are studies suggesting that both are regarded as cost-effective compared to GPs	• Cost-effectiveness is less clear for ANPs due to variability in roles compared to PAs
Perceptions		
By colleagues	• Colleagues appreciate the added values and task-sharing from both roles • Both have unclear professional boundaries	• PAs could be subject to more negative perceptions due to a lack of prescribing rights and the fact that the role is not based on existing professions in the UK
By employers	• Employers view both roles as cost-effective and contributing to the work required within the NHS	• ANPs were mostly employed by doctors to undertake specific work, whereas PAs were employed by hospitals in more generalist roles
Self-identity	• Both face lack of clarity with regard to role definition	• PAs seem to encounter more identity crises due to the relative novelty of their roles in the UK
Regulations		
Policy	• Both have lack of formal regulation	• ANPs are credentialed and have prescribing rights, although their prescribing rights are based on nurse prescribers, whereas PAs do not have prescribing rights • ANP regulation is rooted in nurse regulation through the Royal College of Nursing (RCN) • PA regulation is relatively new and is expected to be regulated by the General Medical Council
Governance agencies	• Health Education England has control over the competency framework and education for both roles	• ANPs are governed by RCN, whereas PAs have a Faculty of Physician Associates and are regulated by the General Medical Council

### Competency: education and key skills

Unlike PAs, there is no standard educational route or curriculum for becoming an ANP, despite RCN recommending that ANPs should be educated with a Master's degree [22]. Some ANPs have a masters-level qualification, whereas some ANPs progressed from their existing nursing roles to more specialised roles [8, 23]. PAs, in contrast, are trained through a standardised competence and curriculum framework to meet the requirements of the Faculty of PAs [6].

PAs are expected to have basic medical skills, e.g., diagnosis, treatment, patient management [13, 17, 24–26]. ANPs are required to master nursing skills, and four pillars of advanced practice, including clinical practice, leadership and management, education and research [22]. Skills for ANPs depend more on the specific specialities and the skill mix of ANPs varies across different healthcare contexts because ANPs are specifically recruited to and trained for locally defined roles [11, 27]. PAs are all educated to a similar level, although their skills might vary due to differences in clinical specialities and placements and their skills may further develop depending on specific areas that they are working in [25, 28].

### Career: employment, career progression, and job satisfaction

In terms of employment status, PAs are employed in a wide range of areas, but primarily in secondary care (72% of PAs were employed in secondary care in 2019) [28]. Internship posts for PA positions are paid at band 6 (£32 306–£39 027) and the start pay band for formal PA positions is band 7 (£40 057–£45 839). Experienced PAs can be paid up to band 8a (£47 126–£53 219) [29, 30]. Although the initial orientation of PAs was towards a generalist role in primary care [13, 26], a survey in 2019 of PAs in training in the northwest UK showed a preference for secondary care [31]. The reasons included good educational support, more workload variety, opportunities to learn more specialised healthcare practices and work within teams, possibilities of rotating through different clinical specialities, and better career possibilities [31]. The NHS is encouraging the employment and development of PAs [17, 32]. For example, the National Physician Associate Expansion Programme, which is an innovative project created by the NHS to recruit experienced PAs from the US, placed 27 experienced PAs, including 25 from the US and 2 from the UK, in eight host sites in England from 2016 to 2018 [32, 33]. However, it is predicted that PA recruitment to primary care will likely fall short of the targets [34].

ANPs are employed in band 6/7/8a in the NHS system (band 6, salary from £32 306 to £39 027; band 7, salary from £40 057 to £45 839; band 8a, salary from £47 126 to £53 219) [29] and are employed widely in both primary and secondary care [35–37]. Their jobs are largely context-dependent [35–38] and their job titles vary, including: ANP, lead nurse, matron, nurse practitioner, nurse specialist [11]. The scope of work and employment of ANPs are very much tailored to the needs of local employers [11, 39, 40]. Compared to ANPs, PAs see less variation in clinical roles and job titles, with most PAs being employed in secondary care when compared to ANPs.

There is no defined career progression in clinical roles for PAs beyond the entry-level role. They can become researchers and teachers in universities and hospitals and there is an indication of interest of some PAs to continue to medical school [28]. There are some opportunities for PAs to rotate and advance in different specialities [28, 31, 34, 41]. In contrast, becoming an ANP is part of the career progression of nurses interested in specialisation in a particular clinical area. Further development of ANPs beyond this included: clinical work, such as educating people in self-care and chronic disease, management and leadership, and research [11, 27, 35, 42–44].

There is a lack of research on career satisfaction for both professions. From the limited literature, PAs seem to be generally satisfied with their work [45, 46]. However, the lack of professional recognition and prescribing rights are their main sources of dissatisfaction [45, 46]. One study found that the level of satisfaction for ANPs is comparable to the UK general population as measured by the Short Warwick and Edinburgh Mental Well-being Scale [27]. ANPs derive significant satisfaction from working in primary care. However, some ANPs worry more control and standardised credentialing might limit their flexibility in working [27, 35, 47].

### Effectiveness: patient outcomes and cost-effectiveness

Studies found that consultations with PAs result in similar outcomes and processes as GPs when it comes to less complex tasks in primary care and FY2 doctors in emergency departments [48–50]. In primary care, the prescribing pattern and patient outcome of consultations done by ANPs are similar to GPs in home visits, with fewer referrals [51, 52]. Thus, for both roles, limited data currently suggest the patient outcomes are comparable to that of GPs in primary care for less complex tasks.

As salary and related costs of GPs are higher than those of PAs, a study estimated that the cost of a GP consultation in simple tasks exceeded that of a PA by some GBP £6.22 in 2015 [53]. A cost-effectiveness analysis comparing ANPs, and GPs found that the cost of a GP consultation was nearly 60% higher than that of a Nurse Practitioner for a home visit, after allowing for all investment costs and adjusting for length of consultation [51]. The evidence for ANPs is limited due to the variability in their roles. No evidence was found in relation to secondary care for the cost-effectiveness of ANPs.

### Perceptions: by colleagues, employers, and self-identity

PAs received a mixed reception from other healthcare professionals. The majority of medical workers viewed PAs in a positive light because PAs can share their workload and make the team work more effectively [28, 34, 54, 55]. However, the need for supervision for PAs created some obstacles as doctors need to invest time in this [13]. When PAs were relatively new and seen as being imported from the US, an attitude of “out-group disdain” from doctors and nurses towards PAs was reported [34]. Furthermore, PAs were sometimes met with hostility and a lack of recognition by nurses and doctors due to “groupishness” [34]. The situation is, however, improving as PAs become more established in the workforce [28, 32, 34, 54, 55]. In contrast, ANPs were mostly welcomed by GPs and doctors who appreciated their added value [38–40]. Some doctors expressed concerns out of unfamiliarity with ANPs or fear that ANPs might be exploited due to the unclear definition of ANP roles [38]. Thus, colleagues appreciate the added value from both professions. However, PAs seem to be more subject to a mixed reception by their work colleagues, due to its relative novelty and a lack of awareness of their role, and the lack of prescribing rights, which limited their ability to work independently of doctors without supervision.

More broadly, PAs are being used to fill the vacancies created by the lack of physicians and, as a result, some hospitals report positive experiences [17, 56–58]. ANPs are, however, employed to improve the quality of care and deliver specific services [38–40]. For organisations, ANPs offer an option to retain the workforce not only by providing nursing expertise in clinical practice, but also by contributing to the overall nursing management and research in the organisation and providing a career advance path for nurses [39]. Employers view both roles as cost-effective and contributing to the work required of the healthcare institution.

### Regulations: policy and governance structures

PA training has been governed by the DoH, The UK Association of Physician Associates (UKAPA), and Health Education England (HEE) [13, 25]. There is a lack of formal regulation for PAs to practise in the NHS despite discussion for 20 years [17]. In 2019, the Department of Health and Social Care called for formal regulation of PAs and the General Medical Council (GMC) is expected to begin this in summer 2023, which may pave the way for PAs’ prescribing rights [59]. ANPs are governed by the Royal College of Nursing (RCN), with HEE offering governance for their training [60]. ANPs have also experienced a lack of formal regulation. Although ANPs have been around since the 1980s, credentialing only began in 2016 and it is not compulsory [27]. The definition of ANP roles and governance was largely employer-led [27, 38]. RCN introduced the credentialing framework in 2018, but the framework only serves as guidance [27]. ANPs have prescribing rights based on becoming nurse prescribers, a function open to other nursing cadres.

## Discussion

### PAs and ANPs in the workforce

This review has highlighted that MLPs, including PAs and ANPs, are key components of the UK’s long-term plan for health services. With their numbers growing fast, MLPs are going to become significant contributors to the NHS workforce. PAs can serve an important role in improving efficiency in GP practices and contribute to team-based secondary care [57]. ANPs are shown to be able to reduce referral, share the work of consultant doctors, and support junior staff [8]. ANPs are also easily accessible for patients [43]. Both contribute to the workforce and address physician shortages. They can provide a consistent presence in the medical team as compared to medical students or trainees on rotation, thereby providing potential for improving continuity of care and training and handover to rotating medical trainees.

### Better integration of PAs and ANPs

The key question is how to better integrate these MLPs into the medical workforce. After reviewing the key themes of competency, career development, effectiveness, perceptions, and regulation of PAs in comparison with ANPs, we observed several barriers and enablers for the integration of PAs in the NHS workforce.

Firstly, recruitment initiatives such as work experience, apprenticeships and training centred upon attracting MLPs can facilitate their integration and employment in the NHS, through improving the socialisation, familiarity and acceptability of the MLP roles by creating greater awareness of these professions. The National Physician Associate Expansion Programme demonstrated that getting to know PAs will make the hospital trusts more likely to employ more PAs and include them in their development strategy [32]. Policy-level initiatives to encourage NHS Trusts to employ PAs can also help PAs become desirable in the workforce [17, 32].

Secondly, the absence of prescribing rights is a major concern for PAs and limits their ability to work independently and efficiently [17]. The need for supervision resulting from no prescribing rights also makes other medical workers view PAs as less productive [13]. Lack of prescribing rights is also one of the main sources of dissatisfaction for PAs [45, 46]. The regulation from GMC in summer 2023 may lead to prescribing rights for PAs [59], which could, in turn, put PAs in a better position within the workforce hierarchy and facilitate absorption and integration into the NHS.

Thirdly, awareness from colleagues is important in the integration. PA is a relatively new professional in the UK although it is well-established in the US. “Out-group disdain” could exist and a lack of recognition from nurses and doctors due to “groupishness” was mentioned [34]. ANPs, by contrast, are welcomed given their role is well-grounded in the nursing profession [38, 39]. Studies have shown that more exposure to and familiarity with PAs can help raise awareness and may facilitate inter-professional work [28, 32, 55]. Hospitals might be able to facilitate the integration process by educating the medical workers on the role of PAs and ANPs and the scope of their practice.

Fourthly, professional boundaries and role definition are concerns in the integration. PAs have been reported to experience frustration due to lack of recognition and experience an identity crisis [28, 31, 34, 61]. ANPs, in contrast, view themselves as “mega-nurses” rather than “mini-medics” because they emerged from the nursing profession [40, 43, 44]. Some ANPs mentioned a lack of clarity with definitions of their roles [38, 43, 44]. Lack of clarity of role definition undermines the job satisfaction of both MLP roles [35, 46] and makes them subject to potential exploitation, e.g., to take the work beyond their competency and scope of work [38]. In terms of professional boundaries, PAs spanned the boundaries of medicine and nursing in that they can support doctors and also at times support nurses, for example, patient management, and PAs are seen to be collaborators to both doctors and nurses [13, 17]. ANPs are therefore seen as complementary to medical practice instead of a form of substitution. However, ANPs may pose a threat to traditional nursing [40]. Advanced practice roles not only change the nature of healthcare workers, but also reconstruct professional boundaries [62].

### Strengths and limitations

This review provides a comprehensive picture of the literature on PAs and ANPs in the NHS workforce and discusses the ways that we can improve their integration. The study has immediate policy relevance given GMC is now preparing to regulate PAs. The study has several limitations. Firstly, we did not extensively search grey literature although we felt the amount of information from the literature allowed us to achieve our aims. This may lead to an incomplete picture of the situation. Secondly, the data extraction process was done by a single investigator. Although the extraction process and results have been extensively discussed among all the co-authors, bias could still exist. Thirdly, due to the scope of the research and lack of literature, this study did not present other MLPs, such as medical support workers introduced recently or anaesthesia associates. Lastly, we used established methodology for conducting scoping reviews, we therefore did not include formal quality appraisal of studies in our review. While this is a limitation of scoping reviews, our aim was to synthesise the literature and future systematic reviews exploring a sub-topic of this review might investigate quality of studies in more depth.

## Conclusions

PAs and ANPs are going to become a significant part of the NHS workforce. The current study investigated the career development, competency, effectiveness, perceptions, and regulation of PAs and ANPs in the UK. We discussed potential ways to better integrate them into the workforce, including initiatives from the NHS, prescribing rights, better awareness, and clearer role definitions. Further research can include more MLPs, including medical support workers, and provide a more complete picture of MLPs in the UK. Furthermore, studies focusing on international comparisons between the UK and other countries could be conducted.

## Supplementary Information

**Additional file 1**: Search strategy, databases, and results.

**Additional file 2**: Overview of the Included Studies: Physician Associate (ordered according to the year of publication).

**Additional file 3**: Overview of the Included Studies: Advanced Nurse Practitioners (ordered according to the year of publication).

## Data Availability

All data generated or analysed during this study are included in this published article and its supplementary information files.

## Abbreviations

MLPs: Mid-level practitioners
PAs: Physician associates
ANPs: Advanced nurse practitioners
NHS: National Health Service
RCN: Royal College of Nursing
GMC: General Medical Council
HEE: Health Education England
UKAPA: The UK Association of Physician Associates
DoH: The Department of Health
