# Supplementary material for: The roles of physician associates and advanced nurse practitioners in the National Health Service in the UK: a scoping review and narrative synthesis
Source: Hum Resour Health. 2022 Sep 15;20:69. doi: 10.1186/s12960-022-00766-5 (PMC9479410; doi:10.1186/s12960-022-00766-5)
Supplement: Supplementary file 1 — Additional file 1: Search strategy, databases, and results. [file 12960_2022_766_MOESM1_ESM.docx]

***Appendix 1. Search strategy, databases, and results^*^***

| Search Terms | Results | | | | |
| --- | --- | --- | --- | --- | --- |
|  | Embase | Medline | PubMed | CINAHL | Cochrane |
| # 1 exp Physician Assistants/ | 6,477 | 6,013 | 6,013 | 3,573 | 63 |
| # 2 Physician Assistant$.tw. | 4,841 | 3,837 | 7,816 | 16,657 | 5,568 |
| # 3 physician associate$.tw. | 209 | 189 | 197 | 216 | 16,641 |
| # 4 Medical Assistant$.tw. | 1556 | 990 | 991 | 737 | 21,818 |
| # 5 Allied Health Personnel. tw. | 1,556 | 990 | 12,356 | 2,636 | 1,243 |
| # 6 (mid level adj3 provider$).tw. | 523 | 259 | 55 | 191 | 1,359 |
| # 7 Paramedical Practitioner&.tw. | 1 | 1 | 0 | 7 | 125 |
| # 8 exp Nurse Practitioners/ | 22,630 | 18,392 | 18,391 | 19,187 | 310 |
| # 9 exp Advanced Practice Nursing/ | 2,039 | 1,874 | 1,873 | 11,129 | 33 |
| # 10 (advanced nurse practi* or advanced pract* or anp or anps or apn or apns).tw. | 19,616 | 18,198 | 21,079 | 32,224 | 1186 |
| # 11 exp United Kingdom/ | 351,001 | 378,191 | 378,189 | 325,939 | 6,639 |
| (# 1 OR 2 OR 3 OR 4 OR 5 OR 6 OR 7)  AND # 11 | 222 | 223 | 221 | 367 | 53 |
| (# 8 OR 9 OR 10)  AND # 11, restricted to humans/ article | 1,117 | 1,159 | 1,553 | 2,005 | 34 |

*^*^The search terms were based on Medline and Embase. The terms were modified according to the operators in PubMed, CINAHL, and Cochrane databases.*
