# Supplementary material for: The roles of physician associates and advanced nurse practitioners in the National Health Service in the UK: a scoping review and narrative synthesis
Source: Hum Resour Health. 2022 Sep 15;20:69. doi: 10.1186/s12960-022-00766-5 (PMC9479410; doi:10.1186/s12960-022-00766-5)
Supplement: Supplementary file 2 — Additional file 2: Overview of the Included Studies: Physician Associate (ordered according to the year of publication). [file 12960_2022_766_MOESM2_ESM.docx]

**Appendix 2 Overview of the Included Studies: Physician Associate (ordered according to the year of publication)**

| No | Ref | Study Design | Study Subject | Research Aim | Main Observations |
| --- | --- | --- | --- | --- | --- |
| [1] | Reedy et al., 1980 | Qualitative study | A PA student from US working at a GP practice in Sonning Common, Oxfordshire | To describe the first PA’s working experience in Britain and explore the feasibility of introducing PAs to the British health system. | - No legal or professional barriers existed for an American PA to work in the UK. - The success of PA depended on the induction of the PA to the staff, preliminary assessment of the PA by the whole practice, and patients’ preparation for the PA’s involvement in their cases. |
| [2] | Cox, 2001 | Review | ANPs and PAs in the US and the UK | To introduce PAs and ANPs in the US and the UK and compare their differences. | - ANPs have prescribing rights while PAs do not. - ANPs generally have 2 years of training compared to the 3-year training of PAs. - ANPs’ education, certification and licensure are standardized, whereas the standardisation for PAs does not exist. |
| [3] | Hutchinson et al., 2001 | Review and opinion | PAs in the US and the UK | To review the impacts of PAs for the US system and discuss their potentials in the NHS. | - PAs make a significant contribution to the US healthcare system. - PAs are in a biomedical model and do not perform nursing duties. - Introduction of US-style PAs to the UK have the potential to reduce medical staffing difficulties but might not be able to remove professional boundaries. |
| [4] | Cawley & Hooker, 2003 | Editorial | PAs in the US and international comparison | To discuss the US experience of PAs and the lessons for other countries, including the UK | - PAs are valuable for addressing workforce shortages. - Some systems may see PAs as substitution for nurses, which is a misconception since the scope of practice is different between the two professions. |
| [5] | Roberts et al., 2004 | Qualitative study | Eight PAs in a British hospital | To study junior doctors’ perceptions of PAs | - PAs are generally viewed positively by junior doctors. - Clear definition and close identification with doctors are needed for their success. |
| [6] | Catanzaro & Stewart, 2005 | Review | PAs in the UK | To review the development and role of PAs, their work quality, and potential impacts in the UK. | - PAs imported from the USA have made significant contribution to the British health system. - PA-training programmes are being developed in the UK. - PAs have the potential to address workforce shortage in the UK and improve healthcare quality |
| [7] | Paniagua & Stewart, 2005 | Review | PAs in the UK | To examine the history, roles, challenges, perceptions of PAs in the UK and describe a training programme. | - PAs could exacerbate interprofessional rivalries, especially with nurses. - The framework of PA-training in the UK is based on international experiences including the US. - The role can attract people who do not want to be nurses and doctors into healthcare. - A master’s programme to train PAs has started by Wolverhampton University. |
| [8] | Shepherd & Armitage, 2005 | Editorial | PAs in the UK | To discuss the role, training, assessment and regulation of medical care practitioner in the UK | - Shortage in the medical workforce has led to the introduction of medical care practitioner in the UK. - The NHS Modernisation Agency’s Changing Workforce Programme proposed expansion of professions. - Medical care practitioner, including PAs, was formed in the UK with novel regulation, training, and assessment approaches. |
| [9] | Legler et al., 2007 | Review | PAs globally | To discuss PA model in the US and internationally | - PA education in the US is efficient and flexible and could be implemented in other nations. - There is a rising interest globally for PAs to address workforce shortages. - Patients have a high degree of acceptance and satisfaction with PAs. |
| [10] | Wilkinson, 2007 | Review and opinion | Anaesthesia practitioner in the UK | To discuss the history and the role of anaesthesia practitioner in the UK | - Modernisation Agency of England set up ‘New Ways of Working in Anaesthesia’ programme in 2003 to introduce non-physician anaesthesia. - Training of anaesthesia practitioner in the UK is run by Royal College of Anaesthetists for 27 months. - Opponents of the role cited patient safety and challenge to medical profession as reasons. |
| [11] | Begg et al., 2008 | Review and opinion | PAs in the UK | To review the first five years of PA education in the UK | - The University of Wolverhampton offered the first UK PA education program in 2004, with subsequent programs in other universities. - The department of health is instrumental in steering the development of the role of the PA and the United Kingdom Association of Physician Assistants (UKAPA) is established for moving the profession forward. |
| [12] | Frossard et al., 2008 | Report of qualitative studies | PAs in Australia, Canada, and other nations | To report the situation of PAs in Australia and Canada and draw lessons for other nations. | - A steering committee of stakeholders is needed for PA development. - Legal discussion of the delegation of doctors in supervising PAs is required and clearly defined roles for both PAs and supervisors are needed. |
| [13] | Cheang et al., 2009 | Qualitative study | Patients under the care of medically qualified professionals in the UK | To explore patients’ perceptions of medically qualified professionals, including anaesthetic practitioner, consultant nurse, surgical care practitioners, etc. | - Patients could be confused by the different titles. - Many patients prefer a transparent approach in knowing who their surgeon is. - The majority of the patient would prefer to wait longer for their operation for it to be carried out by a doctor. |
| [14] | Wakefield et al., 2009 | Qualitative study | Assistant practitioner in the UK | To explore the degree of ‘assistive’ in assistant practitioner job descriptions and study the scope of ‘assistive’ in those jobs. | - Discrepancies exist between policy vision and implementation of the assistant practitioner role in practice. - Post-holders to have with accurate and unambiguously written job descriptors. - Currently, the assistant practitioner role is neither seen as a professional role and nor is it a traditional support role. |
| [15] | Gray et al., 2010 | Qualitative study | anaesthesia assistant | To report part of a 29-month evaluation of the first two cohorts of anaesthesia assistant students in England | - In terms of perceptions, role clarity plays a major role in acceptance or resistance of anaesthesia assistants in the theatre and a lack of role clarity can contribute to the barriers to role implementation. - There was a concern about science graduates with no healthcare background in the beginning, but this diminished as these students became more socialised. |
| [16] | Drennan et al., 2011 | Qualitative study | Employers of PAs in England | To explore the motivation of GPs and managers who employed PAs and to investigate the factors that sustained PAs’ employment. | - PAs in this study were all trained in the US. - The motivation for employing PA included increasing capacity to manage patient demand, broaden the skill-mix and cost considerations. - The issues needed consideration included PA’s supervision, lack of a regulatory framework and prescribing rights, and patients’ lack of familiarity. |
| [17] | Farmer et al., 2011 | Mixed methods | 15 US-trained PAs in Scotland | To study the impacts of PAs on health care delivery in NHS Scotland. | - Patients were generally satisﬁed with PAs - PAs appeared to have longer consultations but provided continuity and an educational resource. PAs’ ability was similar to nurse practitioner or GP. - Valued features were generalism, medical background, differential diagnosis and communication. |
| [18] | Hooker & Kuilman, 2011 | Review and qualitative study | PA education in Australia, Canada, the UK, The Netherlands, and the US. | To compare PA education in the five countries. | - Trends in program per capita growth of PA is the largest in the United States, followed by The Netherlands and Canada. - The shortest PA education program length was 24 months and the longest, 36 months. - Outside the US, most education programs are situated in an academic health centre and all non-US programs receive public funding. |
| [19] | Ritsema & Paterson, 2011 | Quantitative report | PAs in the UK | To characterise the PA profession in the UK. | - PA role is starting to develop in the UK, with PAs being used in a wide variety of specialties across the country. - All PAs are trained as generalists and are required to pass a generalist medical examination regularly. - Some specialists discovered the benefits of hiring generalist PAs and providing on-the-job specialty training, which results in a high proportion of the newly graduated UK-trained PAs working in secondary care. |
| [20] | Drennan et al., 2012 | Quantitative report | Sixteen PAs in English primary care | To explore the employment of PAs in English primary care and their contribution. | - Limited numbers of PAs are working within primary care teams in England. - The teams they work in vary from single-handed GP practices to large teams. - A large portion of PAs; work is in providing same day and urgent consultation appointments for patients in primary care. |
| [21] | Halter et al., 2013 | Systematic review | PAs in primary care internationally | To synthesise the evidence of the contribution of PAs within primary care in the UK and around the globe. | - The major of the included studies were from the US. - Around half of PAs were working in the primary care in the US. - Most of PAs’ responsibility is the management of patients with acute presentations, with a tendency to see younger patients. - Studies of costs provide mixed results and the acceptability to patients is consistently found to be high. |
| [22] | White & Round, 2013 | Mixed methods | PAs in intensive care | To evaluate the process of PAs’ introduction into established teams in intensive care | - At first, there was a significant gap between what was anticipated and what the PAs could actually do. The fact that PAs were unskilled in PICU operations, that they would employ other staff members as trainers, and that their pay was out of proportion to their value caused friction soon after they started. - At five months, the beneficial effects of PAs on patient care and the operation of the unit were emphasized by everyone interviewed. The staff discovered that the PAs had adapted successfully and that there was little sign of previous tension. - At ten months, a study found that PAs were managing the majority of PICU procedures, albeit under some supervision. |
| [23] | Williams & Ritsema, 2014 | Quantitative report | Doctors who work with PAs in the UK | To study the satisfaction of doctors with the role of PAs | - Doctors were generally satisﬁed PAs and believed that the addition of the PA was beneficial. - Doctors received positive feedback from patients about the role of PAs. - Participants reported that the unregulated status of PAs impairs their ability to achieve their full potential. |
| [24] | Bampoe, 2015 | Review and Opinion | PA working in anaesthesia in the UK | To describe opinions regarding PAs working in anaesthesia in the UK | - Opinion among doctors has been divided since the introduction of PAs in anaesthesia in the UK. - Some medical anaesthetists saw the increase in numbers of PAs in anaesthesia as a threat to job opportunities and training. - Others reported that PAs can relieve their workload and help address workforce storages. |
| [25] | Drennan et al., 2015 | Quantitative report | PAs and GPs in primary care in the UK | To compare outcomes and costs of consultations by PAs with GPs. | - In simple tasks, there was no significant difference in the processes and outcomes of PA and GP consultations for same-day appointment patients. - PAs could provide an acceptable and efficient addition to the GP workforce. |
| [26] | Freund et al., 2015 | Review | Primary care professionals in six countries | To compare the skill mix, roles and remuneration in the primary care workforce in the United States, Canada, Australia, England, Germany and the Netherlands. | - In the US, Canada, Australia, UK and the Netherlands, nurses are the major non-physician workers in primary care teams - Remuneration is generally determined by the complexity of tasks in most countries under study. - ‘‘Team-care’’ rather than ‘‘delegation’’ is a trend and integration of ‘‘allied health professionals’’ under the supervision of doctors and nurses, but this is often deterred by legislations and traditional role concepts. |
| [27] | Wanyonyi et al., 2015 | Quantitative report | Dental professionals in primary care in England. | To investigate the potential for skill mix use in primary dental care in England. | - Within the NHS, only around 23% of clinical time was spent on “dentist only” tasks, which suggested that most of care undertaken in NHS primary dental service in England could be performed by dental therapists. - The clinical time and workforce requirements can be identified to the detail of patient group and treatment. |
| [28] | Abraham et al., 2016 | Review | Non-medical practitioners in acute care in the UK | To discuss the implementation of non-medical practitioners in NHS acute care in the UK | - There are changes to the delivery of healthcare in the UK and government strategies are guiding the changes. - The planning of the workforce is central to ensuring patient safety, clinical effectiveness and implementation of non-medical practitioner roles for sustainability. - Non-medical PR actioner roles are flexible and can be used in a number of clinical areas and clinical governance, regulation and integration are essential for their success. |
| [29] | de Lusignan et al., 2016 | Mixed methods | PAs in the UK | To investigate the quality of the patient consultation of PAs compared to GPs. | - PAs were arranged with less complex cases and judged as competent and safe, although GP consultations were rated as more competent. - PAs provide a supplementary addition to the medical workforce in GP practices. |
| [30] | Ritsema & Roberts, 2016 | Quantitative report | PAs in the UK | To assess the job satisfaction level of PAs working in the UK | - PAs in the UK are, in general, satisﬁed with their jobs. - PAs are satisﬁed with the relationships with doctors but are less satisﬁed with the degree to which they are able to achieve their full potentials. |
| [31] | Aiello & Roberts, 2017 | Review | PAs in the UK | To review the development of PAs in the UK | - The PA profession is expectd to contribute to the clinical workforce in the UK healthcare economy. - Relevant authorities should communicate the beneﬁts of PAs to the workforce, the governance of PAs and its professional boundaries, and that PAs are complementary to but not a replacement for other medical professionals. |
| [32] | Drennan et al., 2017 | Qualitative study | PAs in the UK | To investigate the perceived effects on professional boundaries and relationships of introducing PAs. | - Professional boundaries become malleable at the micro level of health delivery. - Differing responses between those working at macro, meso and micro levels of the system was created by stratiﬁcation within professional groups, forming perceptions from acceptance to hostility for PAs - The state agency are needed to provide legislations for jurisdictional boundaries, such as prescribing rights. |
| [33] | Halter et al., 2017a | Qualitative study | PAs in the UK | To study the perception of PAs by patients in general practices in the UK. | - Patients’ understanding of the PA role varies and some patient believed the PAs to be doctors. - Most patients reported positive experiences and outcomes of their consultation. - Negative experiences included prescribing delay and when the limits of the PA role were reached, additional GP consultations were needed. - Willingness to consult a physician associate was related to the patient’s assessment of the severity or complexity of the condition and desires for continuity. |
| [34] | Halter et al., 2017b | Quantitative survey | PAs in the UK | To explore the factors influencing PAs’ employment in secondary care in the UK. | - PAs are employed in small numbers in a range of specialties. - PAs were reported to have been employed to fill gaps in medical stafﬁng and support specialty trainees. - Inhibiting factors include shortage of PAs, lack of authority to prescribe, a lack of evidence and colleague resistance. - There is an appetite for employment of PAs, albeit practical and attitudinal barriers still exist. |
| [35] | Howie, 2017 | Review and opinion | PAs in primary care in the UK | To explore continuing professional development for PAs in primary care in the UK | - There are specific educational requirements for PAs which must be met to keep generalist status, including recertification examinations. - These requirements can be fulfilled by using well established pedagogical strategies that are already in use for junior doctors and allied health professionals. |
| [36] | Jackson et al., 2017 | Qualitative study | PAs in primary care in the UK | To explore the inhibiting factors and facilitators to the integration of PAs into the general practice workforce. | - GPs voiced concerns regarding PAs around managing medical complexity and supervision burden, non-prescriber status, and medicolegal implications in routine practice. - Patients were less concerned about specific competencies as long as there was effective supervision and were accepting of a PA role. - ANPs emphasised their own negative experiences entering practice and the need for support to fight stereotypical and prejudicial attitudes. |
| [37] | Rizzolo et al., 2017 | Quantitative survey | PAs in the US and the UK | To understand the motivations for entering PA schools in the UK and the US. | - Flexibility and career change opportunities are rated high for the reasons to choose a PA career. - Income was one of the top choices for the US PA students. - The number of PAs has risen because of demand in both the US and the UK. |
| [38] | Wheeler et al., 2017 | Quantitative survey | PAs in secondary care in the UK | To explore the interprofessional implications of PAs working in secondary care in the UK. | - PAs most frequently work in inpatient wards, with work generally taking place during weekdays. - PAs reported both direct and non-direct patient care and they reported working within a variety of secondary care team staffing compositions. - Line management was largely provided by consultants, but day-to-day supervision varied. |
| [39] | Drennan et al., 2018 | Quantitative survey | PAs in primary care in the UK | To develop a case-mix classification system using data from PA and GP consultation in the UK. | - The newly developed case-mix classification system can assist in classifying the differences in case-mix by professions. - The system could help a fairer assessment of the potential for role substitution and task shifting in primary care. |
| [40] | Henshall et al., 2018 | Qualitative study | assistant practitioner and nurses in the UK | To investigate the role of assistant practitioner from the perspectives of assistant practitioners and registered nurses in the UK | - There is a lack of role clarity and blurring of boundaries between assistant practitioners and registered nurses, with many tasks undertaken by both. - This lack of ownership of ‘nurse-specific’ roles by registered nurses was apparent and clear differences were only encountered about accountability. |
| [41] | Hoggins et al., 2018 | Qualitative study | PA students in the UK | To study the experiences and perceptions of PA students in primary care educational placements in the UK | - Staff showed a lack of familiarity PAs and there was a risk of unrealistic expectations. - In general, staff and students were positive about their experiences, but students expressed anxiety over a large amount of learning in a short timeframe, the perceptions of others, and the reluctance of staff to train them in phlebotomy skills, and uncertainties in their career aspirations for the future. |
| [42] | Snaith et al., 2018 | Quantitative survey | Assistant practitioners in the UK | To explore the role of assistant practitioners across diagnostic imaging in the UK. | - Most assistant practitioners work in general radiography or mammography, with very few in othecr imaging modalities. - Training routes varied across modalities, with most achieving Band 4 on completion of education. Limitations on practice vary between organisations and modalities, with many reporting blurring of the radiographer-AP boundary. |
| [43] | Drennan et al., 2019 | Qualitative study | PAs in the UK | To study the deployment and contributions of PAs in hospital care in England | - A shortage of doctors was the main encouraging factor for employing PAs. - PAs were found to be acceptable, appropriate and safe members of the medical/surgical teams by the majority of doctors, managers and nurses - The lack of regulation and prescribing rights were seen as major problems. |
| [44] | Edwards et al., 2019 | Review | PAs in the UK | To review the history, regulation, etc., of PAs in the UK. | - Organizational culture will likely be very influential on the introduction PAs in the UK. - Policymakers should provide clarity on the role, purpose, scope of autonomy, supervision and responsibility of PAs, and offer education, training and induction specific to the speciality or role. - Knowledge of interprofessional and collaborative practice are necessary. |
| [45] | Hooker et al., 2019 | Review | PAs in the world | To explore working definitions and establish conceptual boundaries around the topic of patient satisfaction with PAs | - PAs are working in 15 countries and their acceptance seems to be successful and satisfaction with their care is similar to physicians. - The review argued that satisfaction is not dependent on the medical provider as long as the demands of the patients are met. |
| [46] | McGregor et al., 2019 | Qualitative study | PA students in the UK | To explore the career aspirations and expectations of student PAs from a PA school in England. | - Dominant personality traits can be seen in the PA students, including early adoptions to system change, valuing ﬂexibility and work–life balance. - Funding support of the PA programmes seems to allow widening participation. - PAs expressed isolation when working in primary care. |
| [47] | Nelson et al., 2019 | Qualitative study | Assistant practitioners, PAs, and practice pharmacists in the UK | To compare how assistant practitioners, PAs, and practice pharmacists were being established in general practice in the UK. | Three key themes demonstrated participants’ perspectives, including:   - Purpose and place of new roles in general practice, involving unclear role definition and tension at professional boundaries - Transition of new roles into general practice, involving risk management, closing training–practice gaps and managing expectations - Future of new roles in general practice, involving demonstrating impact and questions about sustainability. |
| [48] | Ritsema et al., 2019 | Quantitative survey | PAs in the UK | To explore the growth and development of PAs in the UK | - The number of universities offering PA educational programs have risen from 2 programs in 2012 to 29 by the end of 2017, and the PA students are also rising significantly. - Programs only existed in the greater London and West Midlands areas of England in the beginning but in 2017 PAs were being educated in all 4 countries of the United Kingdom. |
| [49] | Roberts et al., 2019 | Mixed methods | Medical education in the UK | To explore the experience of the impact of PAs on postgraduate medical training in the UK. | - Around half of junior doctors reported no overall impact on their training and a third felt that their training was enhanced by the presence of PAs within 6 months of newly appointed PAs. - Small number of trainees experienced dilution of training opportunities. |
| [50] | Szeto et al., 2019 | Review | PAs in the UK | To deepen the understanding of the barriers and facilitators for integrating PAs into the workforce in the UK. | - The integration of PAs into the UK healthcare has been successful in both primary and secondary care. - The barriers to their integration include lack of statutory regulation, poor understanding of the PA role, and uncertain cost-effectiveness. - The facilitators include by the flexibility of the PA role and the safety of PA consultation. - To realise PAs’ full potential, implementation of national and local policies are essential. |
| [51] | Taylor et al., 2019 | Qualitative study | Patients under the care of PAs in the UK | to study patients’ satisfaction with PAs in acute hospital settings | - The interaction between PAs and patients is am example of successful clinician-patient communication. - Patients were generally not familiar with the PA role. Employers of PAs should consider giving attention to informing patients about the roles. |
| [52] | Brown et al., 2020a | Qualitative study | PA students in the UK | To study student PA experiences in primary care and their attitudes to primary care as a career choice. | - Facilitators of student PA choosing primary care trajectories including engaging students with a degree of responsibility in service provision. - Barriers to engagement included ignorance regarding the PA role, and reverence of medical students as a ‘gold standard’. |
| [53] | Brown et al., 2020b | Qualitative study | PA students in the UK | To investigate student PAs’ experiences of clinical training to understand the process of their occupational identity formation. | - Role recognition is essential to community of practice participation and subsequent identity formation, but this is currently lacking for PA students. - Negativity about the role persists and damages student identity formation - PA role models are lacking, causing student PAs to model themselves on medical doctor and identity dissonance is prevalent and exacerbated by career uncertainty. |
| [54] | Drennan & Halter, 2020 | Review | PAs in the UK | To outline the key research evidence and priorities in the development of PAs in the first ten years in the UK. | - key issues that may be of interest to those developing PA research programs included dissemination of the study, perceptions of PAs, service evaluations involving PAs, etc. - Future research of PAs could consider building multidisciplinary teams and networks, collaborating with PA faculty, capitalizing on funding opportunities, disseminating findings to multiple audiences, etc. |
| [55] | Drennan et al., 2020 | Mixed methods | PAs in the acute care in the UK | To study the contribution, efficiency and safety of experienced PAs in acute settings in England | - Experienced PAs were viewed positively by medical and surgical teams, even in services where high levels of scepticism were initially expressed. - The positive contribution included bringing continuity to the medical/surgical team. - The lack of PA regulation with attendant legislated authority to prescribe medicines and order ionising radiation was a hindrance in their deployment and employment. |
| [56] | Halter et al., 2020 | Mixed methods | PAs in the acute care in the UK | To compare the contribution of PAs to the processes and outcomes of emergency medicine consultations with that of foundation year two doctors-in- training. | - PAs in emergency departments in England treated patients with varies of conditions safely - PAs were evaluated as assessing patients in a comparable way to foundation year two doctors- in- training and providing continuity in the team. Patients perceived PAs positively but had poor understanding of the role. |
| [57] | Howarth et al., 2020 | Quantitative survey | PAs in the UK | To understand the early experiences of PAs and impact factors of their engagement in the UK. | - The demographic of PA students is different to that of medical students. - Caring responsibilities of PAs are highly associated with their engagement. - A lack of understanding of the PA role in clinical settings may need to be addressed to better support and develop the PA profession. |
| [58] | Kim & Bloom, 2020 | Review and opinion | PAs in emergency care in the UK | To explore the British experience with deploying PAs in emergency care. | - PAs can be particularly useful in the Clinical Decisions Unit and Ambulatory Care Unit and they have an active role in documenting in patients’ notes when seeing patients together with a senior doctor. - The strength of PAs in emergency department included flexibility, employment stability, and potential for further expansion and development. - The limitations included PAs’ relatively short duration of training, lack of clinical experience, and dependence on clinicians. |
| [59] | Spooner et al., 2020 | Quantitative analysis | Primary care workforce in the UK | To explore the composition of the primary care workforce in England with changing healthcare workers. | - There is regional variation in staff composition across England, with differences persist when calculated as mean full-time equivalent per thousand patients. - Most workers still are employed as long-established primary care roles, with only a small proportion of new types of practitioners, including pharmacists, paramedics, physiotherapists, and PAs. |
| [60] | Shah et al., 2021 | Quantitative assessment | PAs in a community-based mental health setting | To evaluate an enhanced physical health clinic led  by physician associates (PAs) for patients with severe mental illness. | - PAs can be incorporated into mental health multidisciplinary teams in community settings and support the physical health of people with severe mental illness. - Mental health trusts should take PAs into consideration when doing their workforce planning. |

***List of Included Studies: PAs***

1. Reedy BL, Stewart TI, Quick JB: **Attachment of a physician's assistant to an English general practice.** *British medical journal* 1980, **281:**664-666.

2. Cox CL: **Advanced nurse practitioners and physician assistants: what is the difference? Comparing the USA and UK.** *Hospital medicine (London, England : 1998)* 2001, **62:**169-171.

3. Hutchinson L, Pittilo M, Marks T: **The physician assistant: Would the US model meet the needs of the NHS?** *British Medical Journal* 2001, **323:**1244-1247.

4. Cawley JF, Hooker RS: **Physician assistants: does the US experience have anything to offer other countries?** *Journal of health services research & policy* 2003, **8:**65-67.

5. Roberts CM, Proctor I, Forrer M: **An evaluation by doctors in training of a pilot programme of physician assistants.** *Hospital medicine (London, England : 1998)* 2004, **65:**298-301.

6. Catanzaro R, Stewart A: **Can physician assistants be effective in the UK?** *Clinical Medicine, Journal of the Royal College of Physicians of London* 2005, **5:**344-348.

7. Paniagua H, Stewart A: **Medical care practitioners: introducing a new profession into the UK.** *British journal of nursing (Mark Allen Publishing)* 2005, **14:**405-408.

8. Shepherd S, Armitage M: **A new professional in the healthcare workforce: Role, training, assessment and regulation.** *Clinical Medicine, Journal of the Royal College of Physicians of London* 2005, **5:**311-314.

9. Legler CF, Cawley JF, Fenn WH: **Physician assistants: Education, practice and global interest.** *Medical Teacher* 2007, **29:**e22-e25.

10. Wilkinson D: **Non-physician anaesthesia in the UK: a history.** *Journal of perioperative practice* 2007, **17:**162-170.

11. Begg PAP, Ross NM, Parle JV: **Physician assistant education in the United Kingdom: the first five years.** *Journal of Physician Assistant Education (Physician Assistant Education Association)* 2008, **19:**47-50.

12. Frossard LA, Liebich G, Brooks PM, Robinson L, Hooker RS: **Introducing physician assistants into new roles: International experiences.** *Medical Journal of Australia* 2008, **188:**199-201.

13. Cheang PP, Weller M, Hollis LJ: **What is in a name - patients' view of the involvement of 'care practitioners' in their operations.** *Surgeon* 2009, **7:**340-344.

14. Wakefield A, Spilsbury K, Atkin K, McKenna H, Borglin G, Stuttard L: **Assistant or substitute: exploring the fit between national policy vision and local practice realities of assistant practitioner job descriptions.** *Health policy (Amsterdam, Netherlands)* 2009, **90:**286-295.

15. Gray M, Smith F, McKeown D, Donaldson J, Hendry S, Page L, Scholes J: **Integrating physician assistants into the practice setting.** *Nursing management (Harrow, London, England : 1994)* 2010, **17:**23-27.

16. Drennan V, Halter M, Tye C, Levenson R: **Physician assistants in English general practice: A qualitative study of employers' viewpoints.** *Journal of Health Services Research and Policy* 2011, **16:**75-80.

17. Farmer J, Currie M, Hyman J, West C, Arnott N: **Evaluation of physician assistants in National Health Service Scotland.** *Scottish Medical Journal* 2011, **56:**130-134.

18. Hooker RS, Kuilman L: **Physician assistant education: Five countries.** *Journal of Physician Assistant Education* 2011, **22:**53-58.

19. Ritsema TS, Paterson KE: **Physician assistants in the United Kingdom: an initial profile of the profession.** *JAAPA : official journal of the American Academy of Physician Assistants* 2011, **24:**60.

20. Drennan VM, Chattopadhyay K, Halter M, Brearley S, de Lusignan S, Gabe J, Gage H: **Physician assistants in English primary care teams: a survey.** *Journal of interprofessional care* 2012, **26:**416-418.

21. Halter M, Drennan V, Chattopadhyay K, Carneiro W, Yiallouros J, de Lusignan S, Gage H, Gabe J, Grant R: **The contribution of physician assistants in primary care: a systematic review.** *BMC health services research* 2013, **13:**223.

22. White H, Round JE: **Introducing physician assistants into an intensive care unit: process, problems, impact and recommendations.** *Clin Med (Lond)* 2013, **13:**15-18.

23. Williams LE, Ritsema TS: **Satisfaction of doctors with the role of physician associates.** *Clinical Medicine, Journal of the Royal College of Physicians of London* 2014, **14:**113-116.

24. Bampoe S: **Physicians' assistants in anaesthesia: colleagues or competitors?** *British journal of hospital medicine (London, England : 2005)* 2015, **76:**610.

25. Drennan VM, Halter M, Grant RL, Brearley S, Joly L, Gage H, De Lusignan S, Gabe J, Carneiro W: **Physician associates and GPs in primary care: A comparison.** *British Journal of General Practice* 2015, **65:**e344-e350.

26. Freund T, Everett C, Griffiths P, Hudon C, Naccarella L, Laurant M: **Skill mix, roles and remuneration in the primary care workforce: who are the healthcare professionals in the primary care teams across the world?** *International journal of nursing studies* 2015, **52:**727-743.

27. Wanyonyi KL, Gallagher JE, Radford DR, Harper PR: **Alternative scenarios: harnessing mid-level providers and evidence-based practice in primary dental care in England through operational research.** *Human resources for health* 2015, **13:**78.

28. Abraham J, Whiteman B, Coad J, Kneafsey R: **Development and implementation of non-medical practitioners in acute care.** *British journal of nursing (Mark Allen Publishing)* 2016, **25:**1129-1134.

29. de Lusignan S, McGovern AP, Tahir MA, Hassan S, Jones S, Halter M, Joly L, Drennan VM: **Physician Associate and General Practitioner Consultations: A Comparative Observational Video Study.** *PloS one* 2016, **11:**e0160902.

30. Ritsema TS, Roberts KA: **Job satisfaction among British physician associates.** *Clinical medicine (London, England)* 2016, **16:**511-513.

31. Aiello M, Roberts KA: **Development of the United Kingdom physician associate profession.** *JAAPA : official journal of the American Academy of Physician Assistants* 2017, **30:**1-8.

32. Drennan VM, Gabe J, Halter M, de Lusignan S, Levenson R: **Physician associates in primary health care in England: A challenge to professional boundaries?** *Social science & medicine (1982)* 2017, **181:**9-16.

33. Halter M, Drennan VM, Joly LM, Gabe J, Gage H, de Lusignan S: **Patients' experiences of consultations with physician associates in primary care in England: A qualitative study.** *Health expectations : an international journal of public participation in health care and health policy* 2017, **20:**1011-1019.

34. Halter M, Wheeler C, Drennan VM, de Lusignan S, Grant R, Gabe J, Gage H, Ennis J, Parle J: **Physician associates in England's hospitals: a survey of medical directors exploring current usage and factors affecting recruitment.** *Clinical medicine (London, England)* 2017, **17:**126-131.

35. Howie N: **Continuing professional development for Physician Associates in primary care.** *Education for primary care : an official publication of the Association of Course Organisers, National Association of GP Tutors, World Organisation of Family Doctors* 2017, **28:**197-200.

36. Jackson B, Marshall M, Schofield S: **Barriers and facilitators to integration of physician associates into the general practice workforce: a grounded theory approach.** *The British journal of general practice : the journal of the Royal College of General Practitioners* 2017, **67:**e785-e791.

37. Rizzolo D, Leonard DR, Massey SL: **Factors that Influence a Physician Assistant/Associate Student Career Choice: An Exploratory Study of Students from the United States and United Kingdom.** *The journal of physician assistant education : the official journal of the Physician Assistant Education Association* 2017, **28:**149-152.

38. Wheeler C, Halter M, Drennan VM, de Lusignan S, Grant R, Gabe J, Gage H, Begg P, Ennis J, Parle J: **Physician associates working in secondary care teams in England: Interprofessional implications from a national survey.** *Journal of interprofessional care* 2017, **31:**774-776.

39. Drennan VM, Halter M, Grant RL, Joly L, de Lusignan S, Gage H: **Capturing complexity in clinician case-mix: Classification system development using GP and physician associate data.** *BJGP Open* 2018, **2**.

40. Henshall C, Aveyard H, Doherty A, Green H, Westcott L: **The role of the assistant practitioner in the clinical setting: a focus group study.** *BMC health services research* 2018, **18:**695.

41. Hoggins R, Scott-Smith W, Okorie M: **UK physician associate primary care placements: staff and student experiences and perceptions.** *International journal of medical education* 2018, **9:**286-292.

42. Snaith B, Harris MA, Palmer D: **A UK survey exploring the assistant practitioner role across diagnostic imaging: current practice, relationships and challenges to progression.** *The British journal of radiology* 2018, **91:**20180458.

43. Drennan VM, Halter M, Wheeler C, Nice L, Brearley S, Ennis J, Gabe J, Gage H, Levenson R, de Lusignan S, et al: **What is the contribution of physician associates in hospital care in England? A mixed methods, multiple case study.** *BMJ open* 2019, **9:**e027012.

44. Edwards LD, Till A, McKimm J: **Leading the integration of physician associates into the UK health workforce.** *British journal of hospital medicine (London, England : 2005)* 2019, **80:**18-21.

45. Hooker RS, Moloney-Johns AJ, McFarland MM: **Patient satisfaction with physician assistant/associate care: an international scoping review.** *Human resources for health* 2019, **17:**104.

46. McGregor J, Farrell M, Baker P, Burnett K, Armer N, Baines J: **The career aspirations and expectations of student physician associates in the UK.** *Future Healthcare Journal* 2019, **6:**36-40.

47. Nelson PA, Bradley F, Martindale AM, McBride A, Hodgson D: **Skill-mix change in general practice: a qualitative comparison of three 'new' non-medical roles in English primary care.** *Br J Gen Pract* 2019, **69:**e489-e498.

48. Ritsema TS, Roberts KA, Watkins JS: **Explosive Growth in British Physician Associate Education Since 2008.** *The journal of physician assistant education : the official journal of the Physician Assistant Education Association* 2019, **30:**57-60.

49. Roberts S, Howarth S, Millott H, Stroud L: **Experience of the impact of physician associates on -postgraduate medical training: A mixed methods -exploratory study***Clin Med (Lond)* 2019, **19:**4-10.

50. Szeto MC, Till A, McKimm J: **Integrating physician associates into the health workforce: barriers and facilitators.** *British journal of hospital medicine (London, England : 2005)* 2019, **80:**12-17.

51. Taylor F, Halter M, Drennan VM: **Understanding patients' satisfaction with physician assistant/associate encounters through communication experiences: a qualitative study in acute hospitals in England.** *BMC health services research* 2019, **19:**603.

52. Brown M, Laughey W, Finn GM: **Physician Associate students and primary care paradigmatic trajectories: perceptions, positioning and the process of pursuit.** *Education for primary care : an official publication of the Association of Course Organisers, National Association of GP Tutors, World Organisation of Family Doctors* 2020, **31:**231-239.

53. Brown MEL, Laughey W, Tiffin PA, Finn GM: **Forging a new identity: a qualitative study exploring the experiences of UK-based physician associate students.** *BMJ open* 2020, **10:**e033450.

54. Drennan VM, Halter M: **Building the evidence base-10 years of PA research in England.** *JAAPA : official journal of the American Academy of Physician Assistants* 2020, **33:**1-4.

55. Drennan VM, Taylor F, Halter M, Calestani M, Levenson R: **Perceived impact on efficiency and safety of experienced American physician assistants/associates in acute hospital care in England: findings from a multi-site case organisational study.** *JRSM Open* 2020, **11**.

56. Halter M, Drennan V, Wang C, Brearley S, Wheeler C, Gage H, Nice L, Parle J, De Lusignan S, Gabe J, et al: **Comparing physician associates and foundation year two doctors-in-training undertaking emergency medicine consultations in England: A mixed-methods study of processes and outcomes.** *BMJ Open* 2020, **10:**037557.

57. Howarth SD, Johnson J, Millott HE, O'Hara JK: **The early experiences of Physician Associate students in the UK: A regional cross-sectional study investigating factors associated with engagement.** *PloS one* 2020, **15:**e0232515.

58. Kim M, Bloom B: **Physician associates in emergency departments: The UK experience.** *European Journal of Emergency Medicine* 2020, **27:**5-6.

59. Spooner S, Gibson J, Checkland K, McBride A, Hodgson DE, Hann M, McDermott I, Sutton M: **Regional variation in practitioner employment in general practices in England: a comparative analysis.** *The British journal of general practice : the journal of the Royal College of General Practitioners* 2020, **70:**e164-e171.

60. Shah C, Singh P, Matin S, Farrow J, Magon R, Zia A, Tatt-Smith P, Watson C, Smith A: **A physician associate-led clinic for people with severe mental illness in the United Kingdom.** *JAAPA : official journal of the American Academy of Physician Assistants* 2021, **34:**1-6.
