# Supplementary material for: The roles of physician associates and advanced nurse practitioners in the National Health Service in the UK: a scoping review and narrative synthesis
Source: Hum Resour Health. 2022 Sep 15;20:69. doi: 10.1186/s12960-022-00766-5 (PMC9479410; doi:10.1186/s12960-022-00766-5)
Supplement: Supplementary file 3 — Additional file 3: Overview of the Included Studies: Advanced Nurse Practitioners (ordered according to the year of publication). [file 12960_2022_766_MOESM3_ESM.docx]

**Appendix 3 Overview of the Included Studies: Advanced Nurse Practitioners (ordered according to the year of publication).**

| No | Ref | Study Design | Study Subject | Research Aim | Main Observations |
| --- | --- | --- | --- | --- | --- |
| [1] | Atkin & Lunt, 1995 | Qualitative study | ANPs in the UK | To explore the role of continuing training and education from the perspectives of ANPs, GPs, and authorities. | - ANPs utilise a range of education and training opportunities and seem not to be disadvantaged training pathways. - Challenges include the informal arrangements covering training and education. - ANPs training and educational needs will be influenced by the role expected of general medical services and the general development of primary health care services. |
| [2] | Atkin & Lunt, 1996a | Qualitative study | ANPs in the UK | To examines the type of work performed by ANPs and the associate factors. | - The findings suggested that a consensus on the future development of ANPs is unlikely, given the different stakeholders have their own priorities. - The context of ANPs’ role will be shaped by the tensions generated by the different interests and perspectives, and the subsequent organizational and policy initiatives that emerge. |
| [3] | Atkin & Lunt, 1996b | Qualitative study | ANPs in the UK | To study the supervision and management of ANPs in general practice in the UK. | - Managing ANPs poses two different but interrelated issues, namely, the supervision and the day-to-day management and the effective use of ANP potentials in primary health care. - Different stakeholders emphasised different issues reﬂecting their own priorities and backgrounds. |
| [4] | Elcock, 1996 | Review and Opinion | ANPs in the UK | To debate whether ‘consultant nurse’ is an appropriate title for ANPS in the UK. | - Nurses may view many of the principles and processes involved in consultancy as foreign to nursing practice, including the relationship between the consultant and the consultee and the view that the consultant nurse should not have a direct care role. - The term consultant nurse itself has medical connotations and is viewed negatively by many nurses. - Nursing is unique and the term ‘consultant nurse’ cannot replace the term ANP. |
| [5] | Rolfe & Phillips, 1997 | Qualitative study | ANPs in the UK | To investigate the role of ANPs in dementia unit in the UK. | - The ANP was valued as much by other health care professionals as she was by nurses. - In many cases, the ANP role could replace GP for initial assessments and ongoing chnical intervention/ - The role of ANP was perceived as enhancing and extending the professional role of the nurse. |
| [6] | Woods, 1997 | Review and opinion | ANP education in the UK | To present issues for curriculum designers of clinically based advanced practice nurse programmes. | - Consideration needs to be given to the competing forces involved in the conceptualization of different models of advanced practice when determining curriculum content - Advanced nursing practice is the 'advancement of practice and the understanding of nursing', which aims to bring about significant improvements in the quality of patient care. |
| [7] | Roberts-Davis, Nolan, Read, & Gilbert, 1998 | Review and opinion | Advance nursing practice in the UK. | To present the findings of the research project for realizing specialist and advanced nursing practice in the UK. | - A significant number of participants considered ANP role to be both specialist and advanced practice. - There is a need to reflect upon the meaning of the term Nurse Practitioner and how this relates to other innovative roles, which is important when it comes to education preparation. |
| [8] | Barton, Thome, & Hoptroff, 1999 | Review and opinion | ANPs in the UK. | To study the controversy and conflict that has arisen within the nursing and medical professions regarding the emergence of ANPs in the UK. | - Professional and occupational boundary redefinition is essential for the discussion of ANPs. - There is an idea that ANPs may be an evolving and discrete professional group outside the currently accepted professional and occupational. - Both nursing and medicine are faced with challenges in accepting ANPs and the new professional boundary. |
| [9] | Hicks & Hennessy, 1999 | Quantitative survey | ANPs in the UK | To explore a task-based approach to defining the role of the nurse practitioner. | - Both ANPs working in acute and primary care considered advanced clinical activities and research activities to be essential to the role of the nurse practitioner. - The ANPs in primary care reported business and management activities as essential tasks, as compared to the ANPs in the acute sector wh regarded high levels of communication skills, autonomy and risk management to be more important. |
| [10] | Paniagua, 1999 | Qualitative study | ANPs in the US and the UK | To describe the perceptions of a visiting lecturer exchange experience between a UK and a US ANP education program. | - The clinical nurse specialist and nurse practitioner roles were seen as distinct within the USA both in their scope of practice and their work setting. - There seems to be a universal agreement of criteria and standards in both schools albeit academic levels can be - subjective and often difficult to measure and define. |
| [11] | Woods, 1999 | Qualitative study | ANPs in the UK | To examine the issues faced by ANPs in the UK as the new role in practice is being established | - ANPs appear to move through three discrete stages during the transitional process, including a state of `idealism' in the beginning, `organizational governance', where practitioners discover the orientation, goal and delivery of their role is controlled by key stakeholders within the organization and `resolution' where practitioners have been in post for more than 1 year and can be summarized as a state of acquiescence and compromise. |
| [12] | Atkins & Ersser, 2000 | Qualitative study | ANPs in the UK | To present a framework for the education of ANPs in the UK. | - The proposed framework consists of 15 practice attributes that are concerned with development and use of knowledge, personal and professional values, appreciation of wider political contexts, cognitive processes, evaluative and investigative abilities, inter-professional working, leading practice development. - The framework offers some clarity and coherence in specifying the nature and scope of advanced and higher-level practice in the UK. |
| [13] | Barr, Johnston, & McConnell, 2000 | Quantitative survey | ANPs in the UK | To explore patients’ satisfaction with ANPs in a hospital in Northern Ireland. | - Patient satisfaction expressed overall satisfaction with the ANP service and they ‘did not mind who saw me as long as I was seen’, which shows that patients may be happy to see who is available first, whether the NP or the doctor. - Retrospective X-ray audit demonstrated NPs’ ability to interpret X-rays to compare favourably with the ability of the Senior House Officers. |
| [14] | Durie, Roland, Leese, Roberts, & Venning, 2000 | Randomised controlled trial | ANPs in the primary care in the UK. | To compare the cost effectiveness of GPs and ANPs as first point of contact in primary care. | - ANP consultations were significantly longer compared to GPs and ANPs carried out more tests but there was no significant difference in patterns of prescribing or health status outcome. Patients were more satisfied with nurse practitioner consultations. - There was no significant difference in health service costs and the clinical care and health service costs of nurse practitioners and general practitioners were similar. |
| [15] | Wilson-Barnett, Barriball, Reynolds, Jowett, & Ryrie, 2000 | Quantitative survey | ANPs in the UK | To study the activities of nurses who are designated as ANPs in the UK. | - The role for ANPs included assessment of individual and group needs, positive motivation to constantly improve practice, inter-disciplinary and cross agency working for planned change and an ability to identify and prioritise service requirements. - Successful role development such as confidence, commitment and problem-solving powers combined with a positive working environment and supportive managers were recognised as personal attributes for the ANPs. |
| [16] | Ormond-Walshe & Newham, 2001 | Review and opinion | Clinical nurse specialist and the ANPs in the UK | To compare and contrast the role of clinical nurse specialist and ANPs in the UK. | - The main areas of differences between ANPs and clinical nurse specialist included educational requirements, what the role involves, who the client is and whether the role encroaches on a doctor's role. - Level of academia, the intersection with the medical profession's role, direct care of patients and certain specialities are useful markers to distinguish clinical nurse specialists and ANPs but the boundary is unclear. |
| [17] | Williams, McGee, & Bates, 2001 | Mixed methods | Senior nursing roles in the UK | To explore the aspects of work by senior nursing roles in the UK. | - There is a wide range in the grading of senior nursing posts, particularly among specialist/ advanced nurses. - Similar amounts of time to undertaking clinical practice, management, research, consultancy and education were devoted by the ward managers and specialist/advanced nurses. - Generally, there is a positive view of senior nursing roles by other staff in the Trust. |
| [18] | Pearson & Peels, 2002 | Review and opinion | ANPs in the world | To provide an international perspective on advanced nursing. | - Deﬁnition, regulation and recognition of advanced nurse practitioners appeared to the major issues of introduction ANPs. - The impact of the ANP role, including how these expanded roles affect the health care system, and the people working within it, and using its services, is a major consideration. |
| [19] | Carnwell & Daly, 2003 | Qualitative study | ANPs in primary care in the UK. | To investigate the current role of ANPs in primary care in the West Midlands region of the UK | - Between disciplines, there are differences in practice's focus and character, and ANPs who are practice nurse have proficiency in advanced practical patient assessment and diagnosis, which leaves little room for strategic development, at the extreme practice end of the practice-strategic continuum. - At the strategic end of the practice strategy continuum, ANPs can talk on the work of Health Visitors and District Nurse, but they do so in a distinct way, and they participate in multi-agency collaboration, practice development, and policy formulation because they are concerned with community and public health. - District nurses interact with specific patients/carers and the nursing team and their involvement in strategic developments tends to centre on patient care issues like protocol and practice developments. |
| [20] | Daly & Carnwell, 2003 | Review and opinion | Elementary, specialist and advancing nursing practice in the UK | To review and clarify the different emerging nursing roles in the UK. | - The borders between inter- and intra- professional activities are blending more and more as nursing practice diversifies more than ever before. - It is challenging to define the distinct nursing practice levels and roles, and there are still ongoing conceptual disagreements. However, there is a large body of literature that supports the usefulness of new nurse positions in transforming the delivery of healthcare and in providing high-quality care, culminating in recent large-scale randomized controlled trials. |
| [21] | Gerrish, McManus, & Ashworth, 2003 | Qualitative study | Master’s level nursing education in the UK. | To explore the features of Master’s level nursing education. | - The aspirations of nurse lecturers for the future of nursing to be realized by their master's graduates seem sensitive to the current professional climate, particularly the requirement of establishing a legitimate and clear relationship with neighboring professions, such as medicine, on the basis of credibility and competence. - The interviewers' descriptions of nursing, however, did not give it the air of confidence sometimes associated with well-established professions. It also hasn't taken a firm stand as a "new profession." and it seems to be receptive to management and governmental orders. |
| [22] | Bird & Kirshbaum, 2005 | Review | ANP in cancer care in the UK | To explore the role of the clinical research nurse in cancer care in the UK. | - The definition of advanced practice is not determined by the role itself but by the level of skills and the role of a clinical research nurse allows for practice above the level of first registration. - Clinical research nurses may engage in advanced practice, which offers a higher level of career advancement and may make the transition between research and clinical practice easier. This might promote research staff awareness, recruitment, and retention in the field of cancer care and other specialties. |
| [23] | Fairley, 2005 | Review and opinion | Nurse consultant in critical care. | To present how a critical care nurse consultant’s clinical role has evolved within a surgical high dependency unit in a hospital in the UK. | - ANPs were not first thought of as acquiring and using the technical skills typically performed by doctors, but rather as a possible fusion of medicine and nursing where holistic nursing assessment is mixed with symptom-focused physical examination. - Due to insufficient medical coverage and a shortage of senior nurses, which could cause incorrect or delayed actions, the role of ANPs evolved. As a result, the primary responsibilities of the position included ensuring the delivery of high-quality care, reducing risk, and directing therapy to get the intended patient outcome. |
| [24] | Hughes, 2005 | Review and opinion | Advanced practice roles in primary care in the UK | To present the policy driver behind three advanced practices roles (first contact care practitioner, medical care practitioner, ANPs) in primary care in the UK. | - Meeting the specialized learning and development needs of healthcare practitioners who take on these new tasks will be difficult for both education and health care. - In the new NHS, work-based learning models can be a key component of the infrastructure that supports role redesign and service improvement, and specific organizational changes are a requirement for service innovation. |
| [25] | Barton, 2006 | Qualitative study | ANP students in the UK | To present findings from a program on the experiences of students, teachers and clinicians involved in a nurse practitioner degree programme | - The ANP students’ experience of the development of the role led to considerations of the evolution of new career structures and identities. - The development of advanced clinical nursing is dependent on the cooperation of clinical nurses, educators, managers, doctors and politicians. - Strategic policy is essential for the development of a professional clinical nursing career framework. |
| [26] | Norris & Melby, 2006 | Mixed methods | ANPs in acute care in the UK | To investigate the views of medical professionals working in emergency departments on the introduction of the acute care nurse practitioner service in the UK. | - The difficulties with the autonomy and other related inter-professional disputes with the function of the ANP appear to be centered on the doctors' reluctance to enable nurses to practice some more advanced abilities. - The necessity for the ANP was identified by both nurses and doctors, however unless this is addressed prior to the introduction of such advanced practitioners, the blurring of lines between doctors and nurses may result in inter-professional conflict. |
| [27] | Williams & Jones, 2006 | Qualitative study | ANPs in the UK | To explore patients’ assessment of the care provided by ANPs in the UK. | - Time spent in consultations and time as a valuable resource in patients' lives were themes found in the data. - When patients seek advice on their health, time is important to them, whether it is time to talk about concerns or time saved because problems have been cured, reducing the need for additional visits. - Aspects related to the focus and style of consultations are also crucial. |
| [28] | Barton, 2007 | Qualitative study | ANP students in the UK | To explore ANP students’ social transition experience in the UK. | - The ANP students experience a composite of social and cultural transitions, which is similar to Van Gennep’s rite of passage model. - Although social transition is modelled in the literature, the stages of a rite of passage had universal application. |
| [29] | Curtis & Netten, 2007 | Quantitative estimation | Costs of ANP training in the UK. | To present a methodology of calculating nurse practitioners training cost that incorporates the human capital cost. | - The unit cost of an ANP rises by over 24% when the whole cost of qualifications is taken into account. - When all investment expenditures are taken into account and consultation length is taken into account, a GP consultation costs roughly 60% more than an ANP consultation. |
| [30] | Main, Dunn, & Kendall, 2007 | Qualitative study | ANPs in the UK | To investigate how medical professionals view the present and future roles of ANPs in primary care | - The lack of a professional registry, challenges with training and prescribing, discrepancies between the cultures of medicine and nursing, professional boundaries, etc. are only a few of the practical and cultural obstacles to the integration of the ANP role in primary care. - Concerns about breaching professional boundaries need to be addressed on a practical and cultural level in order to promote the integration of the nurse practitioner position. |
| [31] | Aranda & Jones, 2008 | Review and opinion | ANPs in the UK | To analyze the theoretical underpinnings of the new advanced practice roles and place them within the larger social and cultural shifts occurring in healthcare. | - Beyond empirical descriptions and understandings, new advanced practice positions in community nursing can be conceptualized and placed more productively within larger political, social, and cultural processes that are reshaping and positioning community nursing. - Understanding the ambivalence and ambiguity of these stances from a more theoretically informed standpoint requires acknowledging the complexity of identities and subject positions as being mutually generated through organizational change. |
| [32] | Por, 2008 | Review and opinion | ANPs in the UK | to clarify the idea of advanced nursing practice by critically analyzing changes in UK policy and current discussions in the international literature. | - The idea of ANP has generated a lot of discussion over the past 25 years and gives nurses a lot of possibilities. Our knowledge of the history of advanced practice is limited, and it does not provide any recommendations for urgent practice. - Uncertainty persists over the nature of advanced practice and the role of ANP currently. It is still difficult to assess the influence and results of the intricate advanced practice nursing positions. |
| [33] | Gaskell & Beaton, 2010 | Qualitative study | ANP education in the UK | To explain how inter-professional work-based education (was implemented in a UK postgraduate Advanced Practitioner program. | - Inter-professional work-based education coupled to work-based learning forms the basis of the MSc Advanced Practice offered by the University of Salford. - This level of interprofessional work-based education makes it easier to comprehend colleagues' abilities and knowledge bases as well as the connections among the professionals operating in the UK's health care system. |
| [34] | Trevatt & Leary, 2010 | Quantitative survey | Advanced cancer nursing practice in England, Northern Ireland and Wales. | To perform a census of specialist/advanced cancer nursing practice posts within England, Northern Ireland and Wales including variation in job titles. | - Role descriptions, the availability of specialized positions, and the types of tumors or cancer patients affected by those positions are all highly variable. - Given the significance of cancer incidence, there are also few consultant-level nurses in the field, especially when compared to those in medicine, and the charity Macmillan Cancer Support funds one-third of specialized cancer nursing positions. |
| [35] | Barton & Mashlan, 2011 | Qualitative study | ANPs in acute care in the UK. | To evaluate secondary care ANP-led service in an acute hospital-based elderly care rehabilitation service. | - The ANP team's ability to deliver services was influenced by organizational structure and traditions. Senior management is required to take action on developmental constraints. - The ANP-led rehabilitation program met its objectives and is a developing example of non-medical led treatment. - A team of ANPs and medical consultants served as a role model for future service reform in acute hospital settings. Senior health care managers were still faced with difficulties with the current organizational frameworks. |
| [36] | Brook & Rushforth, 2011 | Review and opinion | ANPs in the UK. | To discuss the model and regulation of nurse practitioners and ANPs in the UK. | - One specific subset of ANPs who were termed nurse practitioners is ignored by the wider model of advanced practice. - The hybrid, quasi-medical nature of nurse practitioners' work, which is crucially characterized by independent medical diagnosis and treatment, makes them unique. - Since there are significant hazards involved in this line of work, there should be no less than a set of standards that are governed at the federal level. These have to cover how NPs are educated, evaluated, and registered in order to primarily protect the public. |
| [37] | Currey, Considine, & Khaw, 2011 | Review and opinion | Nursing practice in the UK. | To propose a new nursing role termed the Clinical Nurse Research Consultant in the UK. | - The Clinical Nurse Research Consultant will close the gap between research and practice and work to support evidence-based clinical practice. - The Clinical Nurse Research Consultant must be a doctorally prepared acknowledged clinical expert, have educational expertise, and possess advanced interpersonal, teamwork, and communication skills in order to carry out the duties of this proposed post. - Clinical nurses will be able to carry out and impart their clinical skills in this role, develop practice through research, and serve as role models for the clinical/research nexus. |
| [38] | Currie & Grundy, 2011 | Qualitative study | Advanced practice in Scotland. | To make reference to the management implications of Scotland's adoption of a national advanced practice succession planning development pathway. | - Managers thought the career path was worthwhile. However, there was little strategic planning to match service needs and individual development. - Despite varying degrees of practical support, managers were typically seen by practitioners as being engaged in their professional development. Managers and practitioners are both concerned about the continuous funding for advanced practice development. |
| [39] | Fleming & Carberry, 2011 | Qualitative study | ANPs in acute care in the UK. | To present the experiences of expert critical care nurses in their transition to the role of ANP within an intensive care unit setting. | - ‘Staying the course to advanced nursing practice’ emerged as the core category to the ANPs’ transition, and four related major categories emerged, including ‘finding a niche’, ‘coping with the pressures’, ‘feeling competent to do’, and ‘internalising the role’. - The situational, developmental, and conceptual meaning processes were all integral parts of the substantive theory outlining the key processes at play. |
| [40] | Fotheringham, Dickie, & Cooper, 2011 | Quantitative survey | Emergency Nurse Practitioner in Scotland. | To study how the role of the Emergency Nurse Practitioner has evolved in Scotland. | - In emergency departments across Scotland, the job of the Emergency Nurse Practitioner has gradually merged into the delivery of general healthcare and is now accepted as routine. - The terms "Advanced Nursing Practitioners" and "Nurse Practitioners" are not always interchangeable, and nursing jobs that are given room to develop naturally take on different levels of practice. |
| [41] | Kate Gerrish, Guillaume, et al., 2011 | Quantitative survey | ANPs in the UK | To determine what influences ANPs' ability to encourage front-line nurses to practice evidence-based medicine. | - ANPs employed diverse sources of information, participated in a variety of activities, and positively impacted the practice of front-line nurses by promoting evidence-based practice. - Few ANPs consider themselves experts in evidence-based practice, and those with Masters degrees believe they are more competent in all areas than those with lower degrees. ANPs' skills in this area vary widely. |
| [42] | Kate Gerrish, McDonnell, et al., 2011 | Qualitative study | ANPs in the UK | To determine the strategies employed by ANPs to encourage clinical nurses to use evidence-based practice. | - ANPs served as knowledge brokers, conducting knowledge management and encouraging the absorption of knowledge, to encourage clinical nurses to use evidence-based practice. - Creating various types of evidence, gathering it to serve as a repository for clinical nurses, synthesizing various types of evidence, translating evidence by analyzing, explaining, and distilling it for various audiences, and disseminating evidence through formal and informal channels were all part of knowledge management. - ANPs encouraged the use of evidence by enhancing clinical nurses' knowledge and abilities through mentoring, instruction, clinical problem-solving, and change promotion. |
| [43] | Melby, Gillespie, & Martin, 2011 | Mixed methods | ANPs in acute care in the UK | To explore the views of staff and patients of a potential emergency nurse practitioner service in an acute care setting in the UK. | - Although there were some reservations about the emergency nurse practitioner's role, functions, and related accountability issues, health professionals and patients largely supported an emergency nurse practitioner service. - The advanced role of the emergency nurse practitioner has the support of other professionals and patients, but there are also potential conflictual issues and barriers that could arise when implementing such roles. These issues can and must be resolved through appropriate consultation before the service is introduced. |
| [44] | Mullen, Gavin-Daley, Kilgannon, & Swift, 2011 | Mixed methods | Non-Medical Consultant role in the UK | To evaluate the Non-Medical Consultant role for nursing and midwifery in the North West of England. | - The Non-Medical Consultant role for nursing and midwifery role is efficient, adaptable, outward-looking, and responsive both inside the company and externally on a local, regional, and national level. - Understanding of the function inside the organization was a major obstacle for the Non-Medical Consultant role for nursing and midwifery. - The tiny workforce of Non-Medical Consultant role for nursing and midwifery may limit the experience of individual organizations in creating and sustaining the function. |
| [45] | Jokiniemi, Pietilä, Kylmä, & Haatainen, 2012 | Systematic review | ANPs in the US, the UK, and Australia. | To analyze and synthesize the literature on a specialized advance practice nursing role in the US, the UK, and Australia. | - The study included nurse consultant in the UK, the clinical nurse specialist in the USA, and the clinical nurse consultant in Australia and in total 42 studies were included. - There are a lot of similarities regarding the roles of the nurse consultant, clinical nurse specialist, and clinical nurse consultant and the differences in the positions seem to be the result of corporate or personal decisions rather than regional differences. - It is feasible to have an international agreement on the concept of advance practice nursing and its sub-roles. |
| [46] | Kennedy et al., 2012 | Systematic review | nurse consultant roles in the UK | Examine the effects of nurse consultant jobs in adult healthcare settings to find metrics that may be used to show how they affect patient and professional outcomes. | - The 36 studies that were included in this review revealed that nurse consultants had a generally good impact on a variety of clinical and professional outcomes, which correspond to the proposed framework of impact. - The results support the challenge of assessing the intricate roles that nurse consultants play and recommend the use of both quantitative and qualitative methodologies. |
| [47] | Williamson, Beaver, Twelvetree, & Thompson, 2012 | Qualitative study | ANPs in acute care in the UK. | To investigate the function of ward based ANPs and the effects they have on nursing practice and patient care. | - The data showed that the ANP is a key player because they are utilizing their extensive nursing knowledge, networks, and insider information about healthcare systems to not only facilitate patient care but also to play a crucial role in enabling nursing and medical practice. - The data revealed several sub-themes, such as improving practice and communication, serving as a role model, supporting the patients' journey, and breaking new ground in the field. - Ward-based ANPs play a crucial and essential role in delivering high-quality, all-encompassing patient care, and their function goes beyond serving as junior doctors' substitutes. |
| [48] | Dalton, 2013 | Qualitative study | ANPs in hospitals in the UK. | To explore the perceptions of ANPs in a hospital setting in the UK. | - Four major themes were identified, including diverse definitions of the ANP role between medical and surgical wards in the hospital at day compared to hospital at night work; role vagueness and ambiguity; communication and education needs; and constraints and barriers. - The study discovered varying perspectives and comprehensions of the ANP's daytime duties at the hospital. On the other hand, the idea of a hospital at night seemed to be accurately understood; the function seemed well-established, organized, and capable of orchestrating the necessary reactions for both medical and surgical procedures. |
| [49] | Fotheringham, 2013 | Qualitative study | ANPs in the UK. | To discover how ANPs employ expert judgment, to acquire skill, and to examine how the learning environment affects the development of judgment. | - Identification of participants who share the supervisory group's competence reveals a highly aspirational group for whom the governance of learning encourages participants to feel confident in asking for assistance rather than in identifying their own learning requirements. - As participants develop their motor abilities, they also learn to integrate into and manage a volatile work environment, which is understood as being governed by the context in which it is set. |
| [50] | Gerrish, McDonnell, & Kennedy, 2013 | Qualitative study | Nurse consultants in the UK. | To create of a framework for assessing the influence of nurse consultants on organizational, professional, and patient outcomes, as well as the identification of related impact indicators. | - Three domains of impact of nurse consultant roles were identiﬁed, including clinical signiﬁcance, professional signiﬁcance and organizational signiﬁcance. - Even though their primary areas of attention varied, all nurse consultants demonstrated some evidence of their influence in all three domains. - There was no overlap among the specific markers of impact for all nurse consultants because of the great diversity of nurse consultant jobs. |
| [51] | Illingworth, Aranda, De Goeas, & Lindley, 2013 | Qualitative study | ANP students in the UK. | To explore the educational experiences of students preparing for and engaging in ANP roles | - Three themes, including "re-inventing roles," "re-creating self," and "re-engaging with learning," are used to describe the various and complicated educational preparation for ANP responsibilities in the community. - Advanced roles need people to resolve disputes across occupational boundaries and use sophisticated and varied sources of knowledge and skills. |
| [52] | Franks, 2014 | Mixed methods | ANPs in public health in the UK. | To explore the contribution of nurse consultants in England to the public health leadership agenda. | - Nurse consultants drive practice, support change, and assess effectiveness using their clinical experience. - They support service-wide service delivery, policy formation, and public health policy implementation within healthcare organizations, reflecting desired competences and enhancing health outcomes. - Two obstacles were found, including the fact that nurse consultants lacked the time or motivation to conduct research, which prevented them from demonstrating the value of their work, and the fact that decision-makers frequently failed to recognize the value of their work due to a lack of interprofessional understanding and support. |
| [53] | East, Knowles, Pettman, & Fisher, 2015 | Quantitative survey | ANPs in the UK | To examine the history, current activities, and needs for future development of ANPs inside a significant NHS Trust in England. | - Even within identical occupations, the poll found significant diversity in job titles, educational backgrounds, and current activities. - The need for further assistance in engaging in professional development activities was noted by certain participants. - The creation of governance, education, and succession planning plans for advanced practice nursing needs to be actively pursued. The UK NHS Trusts should create their own registers of ANPs the absence of state legislation in order to promote better administration, governance, and workforce planning systems. |
| [54] | Jones, Powell, Watkins, & Kelly, 2015 | Qualitative study | Advanced practitioners in nursing, physiotherapy, paramedics in the UK. | To investigate how various professional groups in Wales, UK, perceive advanced practitioners' current work and prospects for the future. | - More "joined-up" thinking, support, and development opportunities between advanced practitioners, managers, senior doctors, commissioners, and educators are necessary for the AP function to flourish. - Meeting ever-increasing complex health needs requires collaboration in the planning and delivery of education, innovation, and service delivery. This will ensure that future advanced practitioners are sufficiently trained and supported to realize their full potential and contribute to the necessary innovations in current models of care delivery. |
| [55] | Kennedy, Brooks Young, Nicol, Campbell, & Gray Brunton, 2015 | Qualitative study | ANPs in palliative care in the UK | To investigate the introduction of ANPs in a specialist, multi-professional palliative care context in the UK. | - The ANP job has the ability to create "new identities," redraw the lines between nursing responsibilities, and highlight the relationship-based components of great nursing work. - Due to its flexible role definitions, the ANP has the potential to improve specialist palliative care service delivery. - Since the co-construction of a new nursing identity is a key component of accepting the position, the setting in which advanced nurse practitioner roles are established is crucial. Advanced nursing roles also need to be defined, defended, and given names. |
| [56] | McDonnell et al., 2015 | Qualitative study | ANPs in acute care in the UK | To explore the impact of ANPs substituting for junior doctors on patients, staff members and organizational outcomes in general hospital care settings in the UK. | - The patient experience, results, and safety were all improved by ANPs. - They strengthened the knowledge, competence, and quality of work life of the personnel as well as the burden distribution and teamwork. - The accomplishment of organizational priorities, aims, and policy development were made possible by ANPs. |
| [57] | Kemp, 2016 | Quantitative survey | ANPs in mass gathering events in the UK | To explore the potential of ANPs in reducing referrals to local health care agencies in mass gathering events in the UK | - The fact that none of the patients were referred because the ANP was able to treat wounds on the spot speaks to the ANP's capacity to evaluate, diagnose, and offer suitable treatments and advise, which eliminates the need for further referral for many patients. - At mass gatherings, a well-qualified and experienced ANP lowers the referral to the local health care resources, ambulance transport to hospital rate, and the referral to hospital rate. |
| [58] | Hill, 2017 | Review and opinion | ANPs in the UK | To explore the development and identity of ANPs in the UK | - Particularly in England, there is still a lack of uniformity in ANP titles, role definitions, and practice areas. - The Royal College of Nursing's credentialing program and the promotion of ANP roles in government strategies and policies both support how nursing's identity has changed and grown over time, moving from executing doctors' orders and providing task-oriented care to degree-educated, independent, and evidence-based clinical practitioners. - With these strategies and resources in place, ANPs' identity will grow stronger, and their job will become crucial to providing comprehensive, patient-centered care of the highest caliber and to achieving organizational goals like decreasing hospital admissions and duration of stay. |
| [59] | Teare, Horne, Mohammed, & Clements, 2017 | Qualitative study | Nursing practitioner roles in out-of-hours services in the UK | To compare and contrast job descriptions for nursing practitioner roles in out-of-hours services in the UK. | - Although there were numerous similarities in skills between all the professions, there is a lack of agreement over the clinical competence and skills required associated to job title. - Clarity is needed in the emerging field of out-of-hours nursing in order to guarantee patient security and high standards of care. |
| [60] | Halliday, Hunter, & McMillan, 2018 | Qualitative study | ANP in the UK. | To present ward staff’s perceptions of the role of the ANP in a ‘hospital at day’ setting | There were found to be four main themes:   - There was evidence that the function was successful in terms of organizational priorities, such as the effect on patient flow, in addition to being clinically effective in the ward. - When medical teams rotate, it was noted that having an ANP based on the ward and serving as a "constant" in the ward area promotes team stability and enhances the ward area's functionality. - Despite the positive aspects that were noted, the third theme revealed that having a ward-based ANP may have an impact on skill development and may cause employees to become less skilled. - It was stated that there is still a general misunderstanding of the ANP's function. |
| [61] | Alotaibi & Al Anizi, 2019 | Review | ANPs in Saudi Arabia and the UK. | To compare the ANP role in Saudi Arabia and the UK. | - Throughout the world, the function of an advanced clinical nurse practitioner is viewed differently. - There is disagreement over their roles in various hospital departments across the world, and Advanced Clinical Nurse Practitioners may undertake a variety of activities, some of which are more challenging than others, which may be perceived by others as confusing. - The four pillars of clinical, leadership, education, and research serve as the foundation for advanced clinical nurse practitioners' roles all around the world. The amount to which each is carried out differs. |
| [62] | Collins, 2019 | Quantitative survey | ANPs in the UK | To evaluate the effectiveness of ANPs undertaking home visits in an out of hours urgent primary care service in England | - ANPs can perform urgent home visits with the same efficiency as GPs and they can achieve clinical results that are on par with GPs in these situations. - In comparison to their GP counterparts, they had more consistent documentation and a lower referral rate to secondary care. |
| [63] | Evans et al., 2020 | Review protocol | Advanced practice in the UK | To present a review protocol on the evidence-based advanced practice in the UK. | - The growth of advanced clinical practice roles across a wide range of non-medical professions is actively encouraged in the UK through NHS workforce transformation programs. - Advanced clinical practice role development, deployment, nomenclature, definition, governance, and educational preparation are now highly variable among professions and settings, which hinders these efforts. - By defining and mapping the present evidence base supporting multi-professional advanced level practice in the UK from a workforce, clinical, service, and patient viewpoint, this evaluation could provide a more consistent approach to workforce development in the UK. |
| [64] | Hooks & Walker, 2020 | Qualitative study | Advanced practice in the UK | To evaluate the advanced clinical practice role's use in acute and primary settings in one English region and to comprehend its enablers and impediments. | - Advanced clinical practice roles demanded autonomous clinical decision-making, with a high degree of self-awareness and individual accountability. These roles also improved the quality-of-service delivery, provided clinical career development, and increased job satisfaction for staff. - There were organizational barriers, such as restricted access to referral systems, as well as disparate pay scales and funding, difficulty gaining access to continuing education and research, lack of agreement on role definition and title due to inconsistent regulation and governance, and disparate pay scales and funding. - Facilitators included supportive colleagues and opportunities for peer networking. |
| [65] | Stewart-Lord et al., 2020 | Mixed methods | Advanced practice in the UK | To explore the backgrounds, qualifications, experiences, and involvement in novel care models of advanced clinical practitioners in the allied health professions. | - The "Being Advanced" theme emphasized that expert practice included leadership, the use of specialist and expert decision-making skills, and self-assured and independent practice. - The diversity of the members' jobs, titles, career options, and development opportunities were highlighted via "career routes." - The advanced clinical practitioners defined their services as quicker, easier to obtain, and offering a better patient journey in the subject "Outcomes of the advanced clinical practitioner job." - The theme "Influencing and transforming" focused on ideas for innovation, networking, and distribution, as well as influencing and altering services. |
| [66] | Wood et al., 2020 | Quantitative survey | ANPs in the UK | To compile a cohort of ANPs from all around the UK and to present the results of the initial questionnaire, which covered demographics, job history, and well-being. | - Over 40 ANP job titles were reported, spread across five pay bands, and there was no relationship between the job title and pay band. - Although participant stress levels were higher than the national average for the National Health Service, participant well-being was not substantially different from that of the UK population as a whole. |
| [67] | Wood, King, Robertson, et al., 2021 | Qualitative study | ANPs in the UK | To explore the sources of satisfaction, dissatisfaction and well-being for ANPs in the UK. | - Four themes emerged from the data, including ‘the advanced nurse role and professional identity’, ‘feeling exposed’, ‘support for the advancement of the role’ and ‘demonstrating impact’. - ANPs express a great deal of discontent with their sense of identity in their roles and anxiety about being alone, both of which have a detrimental effect on their wellbeing. - However, they also reported feeling quite satisfied with their jobs, especially when they were well-supported and understood how special a difference, they were making to the organizations they worked for and the lives of the patients. |
| [68] | Wood, King, Senek, et al., 2021 | Mixed methods | ANPs in the UK | To understand the experiences of APNs in the UK during the 2020 COVID-19 pandemic. | - UK APNs experienced COVID-19-related staffing and resource constraints in primary and secondary care as well as across the country's regions. - If this is not acknowledged and handled, the UK runs the prospect of a new crisis in staff morale and retention because half of the APNs questioned were considering a move in employment. - Additionally, APNs voiced concern about patients not receiving routine care as a result of numerous specialties closing or reducing staffing during the crisis. However, there were also several instances of good practice, positive changes and innovation |

***List of Included Studies: ANP***

1. Atkin K, Lunt N: **Training and education in practice nursing: the perspectives of the practice nurse, employing general practitioner and Family Health Service Authority.** *Nurse education today* 1995, **15:**406-413.

2. Atkin K, Lunt N: **Negotiating the role of the practice nurse in general practice.** *Journal of advanced nursing* 1996, **24:**498-505.

3. Atkin K, Lunt N: **The role of the practice nurse in primary health care: managing and supervising the practice nurse resource.** *Journal of nursing management* 1996, **4:**85-92.

4. Elcock K: **Consultant nurse: an appropriate title for the advanced nurse practitioner?** *British journal of nursing (Mark Allen Publishing)* 1996, **5:**1376-1381.

5. Rolfe G, Phillips L: **The development and evaluation of the role of an advanced nurse practitioner in dementia -- an action research project.** *International Journal of Nursing Studies* 1997, **34:**119-127.

6. Woods LP: **Conceptualizing advanced nursing practice: curriculum issues to consider in the educational preparation of advanced practice nurses in the UK.** *Journal of advanced nursing* 1997, **25:**820-828.

7. Roberts-Davis M, Nolan MR, Read S, Gilbert P: **Realizing specialist and advanced nursing practice: a typology of innovative nursing roles.** *Accident and emergency nursing* 1998, **6:**36-40.

8. Barton TD, Thome R, Hoptroff TM: **The nurse practitioner: redefining occupational boundaries?** *International Journal of Nursing Studies* 1999, **36:**57-63.

9. Hicks C, Hennessy D: **A task-based approach to defining the role of the nurse practitioner: the views of UK acute and primary sector nurses.** *Journal of advanced nursing* 1999, **29:**666-673.

10. Paniagua H: **Advanced nursing practice from a cross-cultural perspective.** *British journal of nursing (Mark Allen Publishing)* 1999, **8:**724-729.

11. Woods LP: **The contingent nature of advanced nursing practice.** *Journal of advanced nursing* 1999, **30:**121-128.

12. Atkins S, Ersser SJ: **Education for advanced nursing practice: an evolving framework.** *International journal of nursing studies* 2000, **37:**523-533.

13. Barr M, Johnston D, McConnell D: **Patient satisfaction with a new nurse practitioner service.** *Accident and emergency nursing* 2000, **8:**144-147.

14. Durie A, Roland M, Leese B, Roberts C, Venning P: **Randomised controlled trial comparing cost effectiveness of general practitioners and nurse practitioners in primary care.** *British Medical Journal* 2000, **320:**1048-1053.

15. Wilson-Barnett J, Barriball KL, Reynolds H, Jowett S, Ryrie I: **Recognising advancing nursing practice: evidence from two observational studies.** *International journal of nursing studies* 2000, **37:**389-400.

16. Ormond-Walshe SE, Newham RA: **Comparing and contrasting the clinical nurse specialist and the advanced nurse practitioner roles.** *Journal of nursing management* 2001, **9:**205-207.

17. Williams A, McGee P, Bates L: **An examination of senior nursing roles: challenges for the NHS.** *Journal of Clinical Nursing (Wiley-Blackwell)* 2001, **10:**195-202.

18. Pearson A, Peels S: **Advanced practice in nursing: international perspective.** *International Journal of Nursing Practice (Wiley-Blackwell)* 2002, **8:**S1-4.

19. Carnwell R, Daly WM: **Advanced nursing practitioners in primary care settings: An exploration of the developing roles.** *Journal of Clinical Nursing* 2003, **12:**630-642.

20. Daly WM, Carnwell R: **Nursing roles and levels of practice: A framework for differentiating between elementary, specialist and advancing nursing practice.** *Journal of Clinical Nursing* 2003, **12:**158-167.

21. Gerrish K, McManus M, Ashworth P: **Creating what sort of professional? Master's level nurse education as a professionalising strategy.** *Nursing Inquiry* 2003, **10:**103-112.

22. Bird J, Kirshbaum M: **Towards a framework of advanced nursing practice for the clinical research nurse in cancer care.** *Clinical Effectiveness in Nursing* 2005, **9:**161-171.

23. Fairley D: **Discovering the nature of advanced nursing practice in high dependency care: a critical care nurse consultant's experience.** *Intensive & critical care nursing* 2005, **21:**140-148.

24. Hughes J: **Advanced practice roles in primary care: a critical discussion of the policy and practice implications.** *Work Based Learning in Primary Care* 2005, **3:**119-128.

25. Barton TD: **Nurse practitioners - or advanced clinical nurses?** *British journal of nursing (Mark Allen Publishing)* 2006, **15:**370-376.

26. Norris T, Melby V: **The Acute Care Nurse Practitioner: challenging existing boundaries of emergency nurses in the United Kingdom.** *Journal of clinical nursing* 2006, **15:**253-263.

27. Williams A, Jones M: **Patients' assessments of consulting a nurse practitioner: the time factor.** *Journal of advanced nursing* 2006, **53:**188-195.

28. Barton TD: **Student nurse practitioners - A rite of passage? The universality of Van Gennep's model of social transition.** *Nurse Education in Practice* 2007, **7:**338-347.

29. Curtis L, Netten A: **The costs of training a nurse practitioner in primary care: the importance of allowing for the cost of education and training when making decisions about changing the professional-mix.** *Journal of nursing management* 2007, **15:**449-457.

30. Main R, Dunn N, Kendall K: **'Crossing professional boundaries': Barriers to the integration of nurse practitioners in primary care.** *Education for Primary Care* 2007, **18:**480-487.

31. Aranda K, Jones A: **Exploring new advanced practice roles in community nursing: a critique.** *Nursing inquiry* 2008, **15:**3-10.

32. Por J: **A critical engagement with the concept of advancing nursing practice.** *Journal of Nursing Management* 2008, **16:**84-90.

33. Gaskell L, Beaton S: **Inter-professional work based learning within an MSc in Advanced Practice: Lessons from one UK higher education programme.** *Nurse Education in Practice* 2010, **10:**274-278.

34. Trevatt P, Leary A: **A census of the advanced and specialist cancer nursing workforce in England, Northern Ireland and Wales.** *European Journal of Oncology Nursing* 2010, **14:**68-73.

35. Barton D, Mashlan W: **An advanced nurse practitioner-led service - consequences of service redesign for managers and organizational infrastructure.** *Journal of Nursing Management* 2011, **19:**943-949.

36. Brook S, Rushforth H: **Why is the regulation of advanced practice essential?** *British Journal of Nursing* 2011, **20:**996-1000.

37. Currey J, Considine J, Khaw D: **Clinical nurse research consultant: a clinical and academic role to advance practice and the discipline of nursing.** *Journal of Advanced Nursing (John Wiley & Sons, Inc)* 2011, **67:**2275-2283.

38. Currie K, Grundy M: **Building foundations for the future: The NHS Scotland advanced practice succession planning development pathway.** *Journal of Nursing Management* 2011, **19:**933-942.

39. Fleming E, Carberry M: **Steering a course towards advanced nurse practitioner: a critical care perspective.** *Nursing in critical care* 2011, **16:**67-76.

40. Fotheringham D, Dickie S, Cooper M: **The evolution of the role of the Emergency Nurse Practitioner in Scotland: a longitudinal study.** *J Clin Nurs* 2011, **20:**2958-2967.

41. Gerrish K, Guillaume L, Kirshbaum M, McDonnell A, Tod A, Nolan M: **Factors influencing the contribution of advanced practice nurses to promoting evidence-based practice among front-line nurses: Findings from a cross-sectional survey.** *Journal of Advanced Nursing* 2011, **67:**1079-1090.

42. Gerrish K, McDonnell A, Nolan M, Guillaume L, Kirshbaum M, Tod A: **The role of advanced practice nurses in knowledge brokering as a means of promoting evidence-based practice among clinical nurses.** *Journal of Advanced Nursing* 2011, **67:**2004-2014.

43. Melby V, Gillespie M, Martin S: **Emergency nurse practitioners: the views of patients and hospital staff at a major acute trust in the UK.** *Journal of clinical nursing* 2011, **20:**236-246.

44. Mullen C, Gavin-Daley A, Kilgannon H, Swift J: **Nurse consultants 10 years on: an insight to the role for nurse managers.** *J Nurs Manag* 2011, **19:**820-831.

45. Jokiniemi K, Pietilä A-M, Kylmä J, Haatainen K: **Advanced nursing roles: A systematic review.** *Nursing & Health Sciences* 2012, **14:**421-431.

46. Kennedy F, McDonnell A, Gerrish K, Howarth A, Pollard C, Redman J: **Evaluation of the impact of nurse consultant roles in the United Kingdom: a mixed method systematic literature review.** *Journal of Advanced Nursing (John Wiley & Sons, Inc)* 2012, **68:**721-742.

47. Williamson S, Beaver K, Twelvetree T, Thompson J: **An ethnographic study exploring the role of ward-based Advanced Nurse Practitioners in an acute medical setting.** *Journal of Advanced Nursing* 2012, **68:**1579-1588.

48. Dalton MA: **Perceptions of the advanced nurse practitioner role in a hospital setting.** *British journal of nursing (Mark Allen Publishing)* 2013, **22:**48-53.

49. Fotheringham D: **Confident to seek help: the development of skill and judgement in nurse practitioners. A mixed methods study.** *Nurse education today* 2013, **33:**701-708.

50. Gerrish K, McDonnell A, Kennedy F: **The development of a framework for evaluating the impact of nurse consultant roles in the UK.** *Journal of Advanced Nursing* 2013, **69:**2295-2308.

51. Illingworth A, Aranda KF, De Goeas SM, Lindley PJ: **Changing the way that I am: Students experience of educational preparation for advanced nursing roles in the community.** *Nurse Education in Practice* 2013, **13:**338-343.

52. Franks H: **The contribution of nurse consultants in England to the public health leadership agenda.** *Journal of clinical nursing* 2014, **23:**3434-3448.

53. East L, Knowles K, Pettman M, Fisher L: **Advanced level nursing in England: organisational challenges and opportunities.** *Journal of nursing management* 2015, **23:**1011-1019.

54. Jones A, Powell T, Watkins D, Kelly D: **Realising their potential? Exploring interprofessional perceptions and potential of the advanced practitioner role: a qualitative analysis.** *BMJ open* 2015, **5:**e009740.

55. Kennedy C, Brooks Young P, Nicol J, Campbell K, Gray Brunton C: **Fluid role boundaries: exploring the contribution of the advanced nurse practitioner to multi-professional palliative care.** *Journal of clinical nursing* 2015, **24:**3296-3305.

56. McDonnell A, Goodwin E, Kennedy F, Hawley K, Gerrish K, Smith C: **An evaluation of the implementation of Advanced Nurse Practitioner (ANP) roles in an acute hospital setting.** *Journal of advanced nursing* 2015, **71:**789-799.

57. Kemp AE: **Mass-gathering Events: The Role of Advanced Nurse Practitioners in Reducing Referrals to Local Health Care Agencies.** *Prehospital and disaster medicine* 2016, **31:**58-63.

58. Hill B: **Exploring the development and identity of advanced practice nursing in the UK.** *Nursing management (Harrow, London, England : 1994)* 2017, **24:**36-40.

59. Teare J, Horne M, Mohammed MA, Clements G: **A comparison of job descriptions for nurse practitioners working in out-of-hours primary care services: implications for workforce planning, patients and nursing.** *Journal of clinical nursing* 2017, **26:**707-716.

60. Halliday S, Hunter DJ, McMillan L: **Ward staff perceptions of the role of the advanced nurse practitioner in a 'hospital at day' setting.** *British journal of nursing (Mark Allen Publishing)* 2018, **27:**92-97.

61. Alotaibi T, Al Anizi A: **Literature Review on Comparisons of Advanced Clinical Nurse Practitioner Role in Saudi Arabia and United Kingdom.** *Journal of Radiology Nursing* 2019, **38:**264-271.

62. Collins D: **Assessing the effectiveness of advanced nurse practitioners undertaking home visits in an out of hours urgent primary care service in England.** *Journal of nursing management* 2019, **27:**450-458.

63. Evans C, Poku B, Pearce R, Eldridge J, Hendrick P, Knaggs R, McLuskey J, Tomczak P, Thow R, Harris P, et al: **Characterising the evidence base for advanced clinical practice in the UK: a scoping review protocol.** *BMJ Open* 2020, **10:**e036192.

64. Hooks C, Walker S: **An exploration of the role of advanced clinical practitioners in the East of England.** *British journal of nursing (Mark Allen Publishing)* 2020, **29:**864-869.

65. Stewart-Lord A, Sinclair N, Beanlands C, Woods S, Khine R, Shamah S, Woznitza N, Baillie L: **The role and development of advanced clinical practice within allied health professions: A mixed method study.** *Journal of Multidisciplinary Healthcare* 2020, **13:**1705-1715.

66. Wood E, King R, Allmark P, Senek M, Tod A, Ryan T, Robertson S: **Advanced practice nurses' experiences and well-being: Baseline demographics from a cohort study.** *Journal of nursing management* 2020, **28:**959-967.

67. Wood E, King R, Robertson S, Senek M, Tod A, Ryan T: **Sources of satisfaction, dissatisfaction and well-being for UK advanced practice nurses: A qualitative study.** *Journal of nursing management* 2021, **29:**1073-1080.

68. Wood E, King R, Senek M, Robertson S, Taylor B, Tod A, Ryan A: **UK advanced practice nurses' experiences of the COVID-19 pandemic: A mixed-methods cross-sectional study.** *BMJ Open* 2021, **11:**e044139.
